# Frequency of chimerism in populations of the kelp *Lessonia spicata* in central Chile

**DOI:** 10.1371/journal.pone.0169182

**Published:** 2017-02-24

**Authors:** Alejandra V. González, Bernabé Santelices

**Affiliations:** 1 Departamento de Ciencias Ecológicas, Facultad de Ciencias, Universidad de Chile, Santiago, Chile; 2 Departamento de Ecología, Facultad de Ciencias Biológicas, Pontificia Universidad Católica de Chile. Santiago, Chile; Northwest A&F University, CHINA

## Abstract

Chimerism occurs when two genetically distinct conspecific individuals fuse together generating a single entity. Coalescence and chimerism in red seaweeds has been positively related to an increase in body size, and the consequent reduction in susceptibility to mortality factors, thus increasing survival, reproductive potential and tolerance to stress in contrast to genetically homogeneous organisms. In addition, they showed that a particular pattern of post-fusion growth maintains higher genetic diversity and chimerism in the holdfast but homogenous axes. In Chilean kelps (brown seaweeds), intraorganismal genetic heterogeneity (IGH) and holdfast coalescence has been described in previous research, but the extent of chimerism in wild populations and the patterns of distribution of the genetically heterogeneous thallus zone have scarcely been studied. Since kelps are under continuous harvesting, with enormous social, ecological and economic importance, natural chimerism can be considered a priceless in-situ reservoir of natural genetic resources and variability. In this study, we therefore examined the frequency of IGH and chimerism in three harvested populations of *Lessonia spicata*. We then evaluated whether chimeric wild-type holdfasts show higher genetic diversity than erect axes (stipe and lamina) and explored the impact of this on the traditional estimation of genetic diversity at the population level. We found a high frequency of IGH (60–100%) and chimerism (33.3–86.7%), varying according to the studied population. We evidenced that chimerism occurs mostly in holdfasts, exhibiting heterogeneous tissues, whereas stipes and lamina were more homogeneous, generating a vertical gradient of allele and genotype abundance as well as divergence, constituting the first time “within- plant” genetic patterns have been reported in kelps. This is very different from the chimeric patterns described in land plants and animals. Finally, we evidenced that IGH affected genetic differentiation among populations, showed lower levels of F_ST_ index when we compared holdfast than lamina samples. In the light of this, future studies should evaluate the significance of chimeric holdfasts in their ability to increase kelps resilience, improve restoration and ecosystem service.

## Introduction

Genetic diversity is important for population viability and survival [1, 2, 3, 4]. Greater diversity increases population’s ability to adapt and evolve in changing conditions [5, 6, 7]. At present, studies on genetic diversity include the individual, population and regional levels [8], but often the effect of intraorganismal genetic heterogeneity on individual fitness and the capacity to evolve [9] is not considered.

Intraorganismal genetic heterogeneity (hereinafter IGH) involves the existence of different genomes within a single body [10], changing the traditional notion of individuals characterized by physiological unity, genetic homogeneity, and uniqueness [11]. IGH can arise from two distinct sources of genetic variation known as ‘mosaicism’ and ‘chimerism’ [10, 12]. In this study, we focus on chimerism and its frequency in wild populations of the kelp *Lessonia spicata*. Chimerism occurs when two genetically distinct conspecific individuals fuse together or coalesce, generating a single entity [12]. It occurs in a specific group of organisms including fungi, slime molds [13], grafting plants [14], colonial invertebrates (sponges, hydroids, corals, bryozoans and ascidians [15, 16, 17, 18, 19]), humans, other mammals [15] and macroalgae [12].

Chimerism provides both benefits and costs for organisms [10]. In red macroalgae, the main benefit is an increase in body size, and the consequent reduction in susceptibility to mortality factors, thus increasing survival, reproductive potential and tolerance to stress [20, 21, 22]. For example, laboratory and field studies of the chimeric plant *Gracilaria* and *Mazzaella*, showed a positive correlation of chimerism with survival [23, 24], growth [23, 25], reproduction [21], and environmental stress tolerance [22]. Therefore, chimeric plants showed fitness trait advantages that increase their resilience ability compared to genetically homogeneous organisms. The main cost of chimerism is competition between genetically different cell lineages and the probability of original cell-line replacement by more competitive invasive lines. In invertebrates, this is generally facilitated by cell motility [13] while in red algae, differential growth rates between different cell lines may result in competitive exclusion [25]. The studies of *Gracilaria* and *Mazzaella* have shown however a variation of the above pattern, since somatic fusion of genetically different conspecific individuals may produce a chimeric holdfast. It follows that upright axes of the fused individuals emerge by proliferation and vertical growth from a single cell lineage [25]. Growth rate differences in mixed uprights help to segregate genetically different cell lineages along a given axis. Thus, the resulting axes may be genetically heterogeneous at their basal portions (the chimeric holdfast), but are mostly homogeneous in the more apical portions. This unique pattern of post-fusion growth has the capacity to revert chimerism in the apical portion of the thallus by the differentiation of genetically homogenous erect axes [25] as well as maintaining greater genetic diversity in the chimeric holdfast. In natural populations, in terms of exploitation, this type of holdfast can be considered a priceless *in-situ* reservoir of natural genetic resources and variability.

Recently, holdfast fusion and IGH have been described in many kelps and kelp-like species in Chile [26, 27, 28]. These kelps can be considered ecosystem bioengineers and have enormous social, ecological and economic importance. *In-situ* holdfast fusion in *Lessonia berteroana* has been associated with positive ecological consequences, protecting thalli from benthic herbivore pressures and wave-induced mortality [28, 29, 30]. In the laboratory, holdfast fusion of *Lessonia spicata* (Suhr) Santelices, *L*. *berteroana* Montagne, *L*. *trabeculata* Villouta & Santelices, *Macrocystis pyrifera* (Linnaeus) C. Agardh and *Durvillaea antarctica* (Chamisso) Hariot, follows a general pattern of cellular changes [27], suggesting a convergent morphological process among several algal groups during fusion. Genetic studies in natural populations corroborate the high frequency of plants with more than one genotype in *L*. *berteroana* (62–93%, [26, 28]) and *L*. *spicata* (63–87%, [26]). Similarly, a recent study in two National Reserves (Pingüino de Humbold and Fray Jorge, [31]) evidenced a high frequency (>60%) of *L*. *spicata* plants composed by two or more genetically heterogeneous stipes, but none of these showed mixed tissues in the same stipe. Additionally, chimeric plants exhibited higher reproductive success than non-chimeric ones, suggesting benefits of chimeric conditions in natural populations similar to those described for red algae. Therefore, the available data on kelp and kelp-like species in central Chile suggest a high frequency of IGH in natural populations, but their respective quantification in terms of the relative importance of chimerism in these results has not been critically examined. Often, no distinction has been made between the relative contribution of mosaicism versus chimerism; nor has the potential occurrence of some methodological errors been given consideration.

In Chile, kelp is a major raw material for the alginate and biofuels industry, as well as for invertebrate cultures [32, 33]. The continuous harvesting of kelp along the Chilean coast is likely to reduce, fragment and isolate natural populations. This increased demand has led to the introduction of a management plan that includes a population re-colonization strategy (Statute 20,925). A key factor for the management and restoration of threatened and endangered species (terrestrial and aquatic ecosystems) is the conservation of genetic diversity at the individual, population and regional levels [8]. However, in Chilean kelps, the genetic diversity at either of these levels has still been scarcely studied. Furthermore, the complications that IGH and the presence of chimeric plants represent should also be noted. If, as proposed in this study, chimeric plants effectively constitute a reservoir of genetic diversity that could help in a natural re-colonization process providing locally adapted genotypes, then recognition and quantifications of chimeric plants in natural population under exploitation would be of great importance in helping to preserve genetic diversity.

In this study, we therefore examined the frequency of IGH and chimerism in three harvested populations of *L*. *spicata*. We then measured the location of chimeric tissues in different thallus zones by evaluating whether wild-type holdfast showed mixed tissues with higher genetic diversity than erect axes (stipe and lamina). Finally, we explored the impact of differential genetic diversity in different thallus zones on the traditional estimation of genetic diversity at the population level. This work provides a descriptive framework for understanding genetic diversity at the intraorganismal level in brown macroalgae, representing a new approach to understanding chimerism in natural populations, and increasing genetic knowledge to improve management and conservation programs.

## Material and methods

### Sampling collection

**Fig 1:**
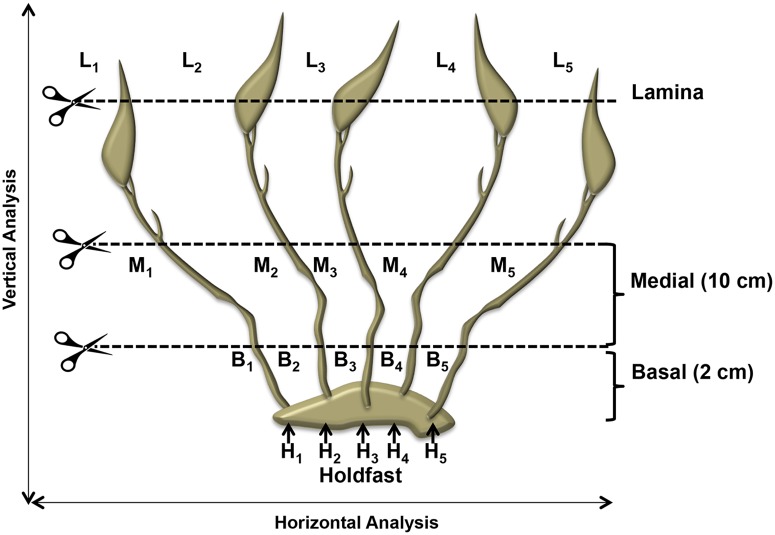
Scheme representing different “within-plant” thallus-zone samples, and the horizontal and vertical analysis used in this study.

Samples of *Lessonia spicata* (Lessoniaceae, Laminariales) were collected from three populations—assigned as Management and Exploitation Areas for Benthic Resources (MEABR)—used as harvesting areas by small-scale fishermen, with annual harvested weight of up to 7100 dry tons [34]. In these populations no specific permission was required for field studies which covered approximately 330 km of the plants’ range of distribution: Pichicuy (PI, 32°20’S-71°27’W) with 6100 dry tons of annual harvested weight. Maitencillo (MA, 32°38’S-71°26’W) with 825 dry tons, and La Puntilla of Pichilemu (LP, 34°22’S-71°00’W) with only 170 dry tons. In each population, we collected fifteen individuals with holdfasts 20–25 cm in diameter, each with 10–15 stipes, 30–40 cm apart from one another. They were extracted during winter (Jun-Jul) from an intertidal platform with similar *L*. *spicata* density (2.3 thalli m^-2^ in Pichicuy, 2.5 thalli m^-2^ in Maitencillo, and 2.1 thalli m^-2^ in La Puntilla). In order to evaluate intraorganismal genetic heterogeneity, we subsampled tissues from the holdfasts (H), basal (B) and medial (M) portions of the stipes, and the apical part of the lamina (L); this was repeated five times for each individual (Fig 1). A total of 900 samples (300 per population) were dried in silica gel for DNA extraction and genetic characterization.

### DNA extraction and genotyping

Genomic DNA from the tissues was extracted using the phenol—chloroform method [35] with modification [36]. The DNA samples were used for microsatellite genotyping (8 microsatellites), following the standard protocols developed for *L*. *spicata* [37] with modification [26]. Amplifications were made using forward primers with a fluorescently labeled M13 tail [38]. PCRs were carried out in 12.5 μL of solution containing 25 ng of template DNA, 0.2 μm of each primer, 0.2 μm of fluorescently labeled (FAM, VIC, PET or NED) M13 universal primer, 100 μm of each dNTP, 2 mm of MgCl2, 1.25 μL 10x PCR buffer, and 0.5 U Taq DNA polymerase (Invitrogen). Cycling conditions consisted of an initial denaturing step of 5 min at 94°C, followed by 30 cycles of 45 s at 94°C, 45 s at the specific temperature [see 37 for details], 2 min at 72°C, and a final elongation step at 72°C for 5 min. For genotyping, 1 μL of the PCR product was added to 22 μL formamide and 0.5 μL LIZ-400 size standards. The mixture was run on an ABI PRISM 3130 XL Genetic Analyzer (Applied Biosystems, Hitachi, Japan) and analyzed using Peak Scanner Software version 1.0 (Applied Biosystems, Foster City California, USA). All samples were re-amplified in order to avoid missing data; deviating samples per plant were re-amplified and re-scored three times to confirm the deviating genotype. All results were recorded manually by two independent observers. Fragment sizes were entered into Microsoft Excel for further analysis.

### Analysis

Micro-Checker software (v 2.2.3) [39] was first used to examine null alleles. We only used a single (the most common) genotype per plant to avoid artificially (sampling pattern) induced deviations from the Hardy—Weinberg equilibrium. In addition, we checked the frequency of each allele in order to avoid the occurrence of false alleles and electrophoresis artifacts [40]. Then, we eliminated loci that showed alleles exclusively observed as heterozygous but not homozygous, and alleles combined solely with another specific allele.

From a total of 45 plants, with 20 samples from different thallus zones, we first estimated different numbers of genotypes by plant, considering a difference of at least one allele in their multilocus genotypes to indicate a different genotype. Secondly, we estimated different numbers of genetic clusters by plants obtained from the Bayesian clustering analysis in STRUCTURE [41]. To this end, the STRUCTURE was run using the default settings (burn-in period of 25000 and 50000 MCMC, admixture model, frequencies correlated, with 5 iterations per K, and K ranging from 1 to 45). The results were then uploaded to a STRUCTURE HARVESTER [42]. The resulting merged data file was then interpreted using the program CLUMPP 1.1.2 [43] and processed by DISTRUCT 1.1 [44]. We repeated these clustering comparisons three times for each population, which contained fifteen plants with 20 samples (N = 300), to visualize and detect differing “within plant” genotypes. Finally, sample relatedness (defined as the probability that two individuals (2n) share an allele due to recent common ancestry [45]) was estimated using the “Queller and Goodnight” relatedness estimator (QG) for all plants with GenAlEx 6.5 software [46, 47]. So, theoretically, if the QG-value was less than 0.25, samples were considered to be unrelated (NR); where QG ranged from 0.25–0.5, they were considered half sibs; if QG ranged from 0.5–0.9, samples were considered similar to full-sibs, but if the QG-value was equal to 1 (maximal similitude among samples), they were considered clones. We considered three levels of analyses: “within plant”, when samples came from the same individual but different thallus zones (H, B, M or L); “among plants”, when samples came from the same population; and “among populations” when samples came from Pichicuy, Maitencillo and La Puntilla.

#### a) Frequency of IGH and chimerism

We estimated the frequency of plants with IGH in natural populations by quantifying the number of plants showing more than one genotype. As we had 20 samples per plant, we considered a plant to be homogenous when all the thallus zones exhibited the same genotype. In contrast, chimerism was estimated by using two different approaches: i) plants that showed different genotypes that diverged in 2 or more different loci. ii) plants that showed more than one genetic cluster [19], obtained from the Bayesian clustering analysis in STRUCTURE, and differing by more than 60% in their cluster assignment probability. Finally, we compared IGH and the frequency of chimerism among populations using a *G-test* [48].

#### b) Genetic diversity at individual and population levels

In order to describe genetic variability at different levels, standard indices of genetic diversity were estimated. Firstly, at the “within-plant” level, we estimated at different thallus zone the total number of different genotypes, the number of genotypes that diverged in 2 or more different loci, and genetic diversity (measured as the number of different alleles and expected Heterozygosity (H_E_) using the GenAlEx 6.5 software [46, 47]). Statistical differences were compared using a one-way ANOVA analysis [48] and GLM [49]. Secondly, a three-level AMOVA [50] analysis was performed using ARLEQUIN v.1.1 [51] considering the “within plant”, “among plant”, and “among population” levels. Significance was tested using 10,000 permutations. Finally, at the population level, we evaluated whether IGH affects traditional genetic estimations. To achieve this, we simultaneously estimated the pairwise F_ST_ index among populations by using independent samples from different thallus zones (H, B, M and L). Population F_ST_ values were estimated for each locus and across all loci according to [52]; pairwise population differentiation (F_ST_) was calculated among all populations using GENETIX v. 4.0 [53] and significance tested using 10,000 permutations. In the same way, we performed principal coordinate analyses (PCoA) via covariance matrices with data standardization using GenAlEx 6.5, in order to visualize changes in the genetic distance between populations using samples from different thallus zones.

## Results

All microsatellites were successfully used for genotyping the kelp *L*. *spicata* without missing data and registered only a maximum of two alleles per locus, suggesting that all samples was genetically uniform. However, we eliminated the data from microsatellite loci LESS2D1 because the MICRO-CHECKER evidenced null alleles, and from loci LESS2D6 because alleles 146 and 148 were never observed as homozygous, suggesting possible electrophoresis artifacts.

In terms of the number of genotypes, we found a total of 171 genotypes in the three studied populations; 54 different genotypes were exclusively found in Pichicuy; 93 occurred in Maitencillo and 24 in La Puntilla (S1 Table). The analyses showed that plants consisted of several genotype combinations ranging from 1 to 17 per plant (Table 1). Most genotypes were exclusive to each plant, but we detected eight different plants sharing the same genotypes: genotype 1 found in PI1 and PI12; genotype 66 found in MA5 and MA15; genotypes 153 and 154 found in LP3 and LP13; genotypes 168 found in LP11 and LP15 (Table 1). The total number of genotypes per plant (Table 2) showed significant differences among populations (F_2,42_ = 10.1; P<0.001), with La Puntilla having the lowest number of genotypes per plant (mean 1.9 ± 0.8) compared to Pichicuy (mean 3.6 ± 1.4) and Maitencillo (mean 6.4 ± 4.8). In the same way, significant differences among populations (G = 64.8, df = 2, *P*<0.001) were found in terms of the number of genotypes that differed in more than 2 loci (Table 2), where Pichicuy had the highest frequency (86.7%) followed by Maitencillo (66.7%) and La Puntilla (33.3%)

**Table 1:** List of different genotypes per plant from the three harvested populations of *L*. *spicata*, at different thallus zones. H: Holdfast, B: Basal stipe, M: Medial stipe, L: Lamina. The asterisk indicates that genotypes showing a QG value lower than 0.25 are considered to be unrelated genotypes.

		Replicate 1			Replicate 2			Replicate 3			Replicate 4			Replicate 5		
*Pichicuy*	Holdfast (H)	B_1_	M_1_	L_1_	B_2_	M_2_	L_2_	B_3_	M_3_	L_3_	B_4_	M_4_	L_4_	B_5_	M_5_	L_5_
PI1	1,1,1,1,1	2	2	2	1	1	1	1	1	1	1	1	1	1	1	1
PI2	3,3,3,3,4	3	3	3	3	3	3	3	3	3	4	4	4	4	4	4
PI3	5,5,5,5,5	5	5	5	6	5	5	5	5	6	5	5	6	7	5	5
PI4*	8*,9,10,10,10	8*	8*	8*	10	10	10	10	10	10	10	10	10	10	10	10
PI5	11,13,11,14,14	12	12	12	14	14	14	14	14	14	15	15	15	14	14	14
PI6	16,16,16,18,16	16	16	16	16	16	16	17	16	16	18	18	18	16	16	16
PI7	19,19,20,22,22	19	19	19	19	19	19	21	21	21	22	22	22	22	22	22
PI8	23,23,23,23,23	23	23	23	23	23	23	23	23	23	24	23	23	23	23	23
PI9*	25,28*,28*,28*,28*	26	27	27	29	30	30	28*	28*	28*	28*	28*	28*	31	31	31
PI10	32,32,32,33,34	32	32	32	32	32	32	32	32	32	33	32	32	32	32	32
PI11*	35*,36,38,39,39	35*	35*	35*	37	37	37	37	37	37	40	40	40	40	40	40
PI12*	1*,42,43,44,44	41*	41*	41*	42	42	42	42	42	42	44	44	44	44	44	44
PI13	45,46,47,48,48	45	45	45	45	45	45	48	48	48	48	48	48	48	48	48
PI14	49,51,49,49,49	50	50	50	50	50	50	49	49	49	50	50	50	49	49	49
PI15	52,52,53,54,52	52	53	53	52	52	52	52	52	52	52	52	52	52	52	52
***Maitencillo***																
MA1	55,56,55,55,57	55	55	55	56	55	55	55	55	55	55	55	55	55	55	55
MA2	58,58,58,58,58	58	59	58	58	58	58	58	58	58	58	58	58	58	58	58
MA3*	60*,61,61,62,61	61	61	61	61	61	61	61	61	61	61	61	61	63	63	63
MA4	64,64,64,65,64	64	64	64	64	64	64	64	64	64	65	64	64	64	64	64
MA5*	66,69*,67,67,67	67	67	68	70	67	67	67	67	67	67	67	71	67	67	67
MA6	72,72,72,72,72	72	72	72	72	72	72	72	72	72	72	72	72	72	72	72
MA7	73,73,74,73,74	73	73	73	73	73	73	74	74	74	73	73	73	74	74	74
MA8*	75,75,75,75,80*	75	75	75	75	75	75	76	77	76	78	79	75	81*	75	75
MA9	82,86,89,91,96	83	84	85	87	88	86	90	91	92	93	94	95	97	98	97
MA10*	99,102,101,104*,101	100	101	101	103	101	101	101	101	101	101	101	101	101	105	101
MA11*	106,106,110,113,117	107	107	106	106	108*	109	110	111	112	114	115	116	118	119	120
MA12	114,122,125,125,129	121	122	123	124	125	121	126	125	125	127	128	129	129	129	129
MA13	130,131,132,130,131	131	131	131	131	131	131	132	132	132	130	130	130	131	131	131
MA14*	133,129,138,139*,138	134	133	133	135	136	137	138	138	138	140	141	135	138	138	138
MA15	142,66,142,146,147	143	143	144	145	145	145	142	142	142	146	146	146	144	144	144
***La Puntilla***																
LP1	148,149,148,149,148	149	149	149	149	149	149	149	149	149	149	149	149	149	149	149
LP2	150,151,152,151,150	150	150	150	152	150	150	152	152	152	151	152	152	150	150	150
LP3	153,153,153,154,153	154	154	154	153	154	154	153	154	154	154	154	154	153	154	154
LP4	155,155,155,155,155	155	155	155	155	155	155	155	155	155	155	155	155	155	155	155
LP5	156,157,158,157,157	156	156	156	157	157	157	157	157	157	157	157	157	157	157	157
LP6	159,160,160,159,149	160	160	160	160	160	160	160	160	160	160	160	160	149	149	149
LP7*	161,162*,163,163,163	161	161	161	163	163	163	163	163	163	163	163	163	163	163	163
LP8	164,164,164,164,164	164	164	164	164	164	164	164	164	164	164	164	164	164	164	164
LP9	165,165,166,165,165	165	165	165	165	165	166	165	165	165	165	165	165	165	165	165
LP10	167,167,167,167,167	167	167	167	167	167	167	167	167	167	167	167	167	167	167	167
LP11	168,163,168,168,168	168	168	168	169	168	168	168	168	168	168	168	168	168	168	168
LP12	170,170,170,170,170	170	170	170	170	170	170	170	170	170	170	170	170	170	170	170
LP13	153,154,154,154,154	154	154	154	154	154	154	154	154	154	154	154	154	154	154	154
LP14	171,171,171,171,171	171	171	171	171	171	171	171	171	171	171	171	171	171	171	171
LP15	168,168,168,168,168	168	168	168	168	168	168	168	168	168	168	168	168	168	168	168

**Table 2:** List of different number of genotypes in different thallus zones, the number of different loci among genotypes, the number of genetic clusters per plant and the mean QG value per plant from the three harvested populations of *L*. *spicata*. The asterisk indicates plants consisting of genotypes with a QG value lower than 0.25, considered to be unrelated genotypes. H: Holdfast, B: Basal stipe, M: Medial stipe, L: Lamina.

	N° of genotypes					N° of different	N° of genetic	QG value
*Pichicuy*	H	B	M	L	Total	loci among genotypes	clusters	mean±DS
PI1	1	2	2	2	2	1	1	0.96±0.05
PI2	2	2	2	2	2	3	1	0.79±0.21
PI3	1	3	1	2	3	1,2	1	0.93±0.10
PI4*	3	2	2	2	3	1,3,4	2	0.69±0.38
PI5	3	3	3	3	5	1,2,3	1	0.82±0.16
PI6	2	3	2	2	3	2,3	1	0.87±0.14
PI7	3	3	3	3	4	1,2	1	0.73±0.23
PI8	1	2	1	1	2	1	1	0.99±0.02
PI9*	2	4	4	4	7	1,2,3,4	2	0.59±0.30
PI10	3	1	1	1	3	1,2	2	0.97±0.04
PI11*	4	3	3	3	6	1,2,3,4	1	0.52±0.34
PI12*	4	3	3	3	5	1,2,3,4	1	0.53±0.36
PI13	4	2	2	2	4	1,2	2	0.79±0.19
PI14	2	2	2	2	3	1,2	1	0.74±0.24
PI15	3	1	2	2	3	1,2	1	0.96±0.04
***Maitencillo***								
MA1	3	2	1	1	3	1	2	0.97±0.04
MA2	1	1	2	1	2	1	1	0.99±0.02
MA3*	3	2	2	2	4	1,3,4	2	0.80±0.25
MA4	2	2	1	1	2	1	1	0.97±0.05
MA5*	3	2	1	3	6	1,3,4	2	0.83±0.28
MA6	1	1	1	1	1	0	1	1.00±0.00
MA7	2	2	2	2	2	1	1	0.94±0.05
MA8*	2	4	3	2	7	1,2,3,4,5	2	0.75±0.26
MA9	5	5	5	5	17	1,2,3	2	0.71±0.15
MA10*	4	2	2	1	7	1,2,3,4	2	0.85±0.18
MA11*	4	5	5	5	15	1,2,3	2	0.67±0.18
MA12	4	5	4	4	10	1,2,3	1	0.80±0.10
MA13	3	3	3	3	3	1,2	1	0.86±0.11
MA14*	4	4	4	4	10	1,2,3,4,5	1	0.70±0.20
MA15	4	5	5	4	7	1,2,3,4	2	0.66±0.22
***La Puntilla***								
LP1	2	1	1	1	2	1	1	0.96±0.05
LP2	3	3	2	2	3	1,2	1	0.94±0.04
LP3	2	2	1	1	2	1	1	0.94±0.05
LP4	1	1	1	1	1	0	1	1.00±0.00
LP5	3	2	2	2	3	1,4	1	0.80±0.26
LP6	3	2	2	2	3	1,3,4	1	0.79±0.26
LP7*	3	2	2	2	3	1,3,4	1	0.78±0.25
LP8	1	1	1	1	1	0	1	1.00±0.00
LP9	2	1	1	2	2	1	1	0.98±0.03
LP10	1	1	1	1	1	0	1	1.00±0.00
LP11	2	2	1	1	3	1,2	2	0.94±0.14
LP12	1	1	1	1	1	0	1	1.00±0.00
LP13	2	1	1	1	2	1	1	0.98±0.03
LP14	1	1	1	1	1	0	1	1.00±0.00
LP15	1	1	1	1	1	0	1	1.00±0.00

**Fig 2:**
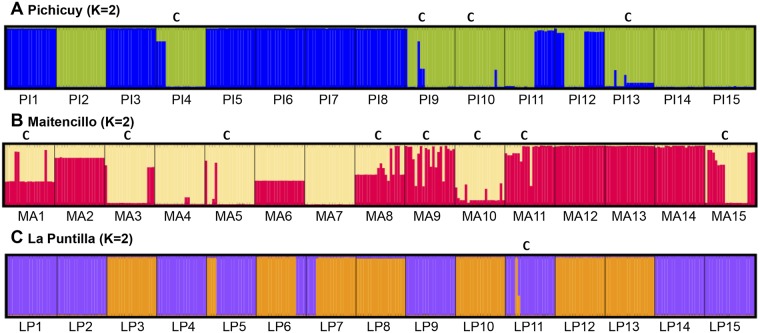
Assignment analyses results from Bayesian clustering analyses using STRUCTURE 2.3, (A) Pichicuy (K = 2, with different colors). (B) Maitencillo (K = 2, with different colors). (C) La Puntilla (K = 2, with different colors). Plants with more than one genetic cluster were considered to be chimeras (C).

In terms of the numbers of genetic clusters per plant obtained from the Bayesian clustering analysis, we found only two genetic clusters (K = 2) in each studied populations (Fig 2). The number of genetic cluster per plant (Table 2) not showed significant differences among populations (Wald statistic = 1.26, df = 2, *P*<0.001), but La Puntilla having the lowest number of genetic cluster (mean 1.0 ± 0.2) compared to Pichicuy (mean 1.3 ± 0.4) and Maitencillo (mean 1.5 ± 0.5). We found that two genetic cluster occurred only in chimeric plants that showed genotypes with 2 to 5 different loci, which in most of cases was a higher threshold than the other approach used in this study.

**Fig 3:**
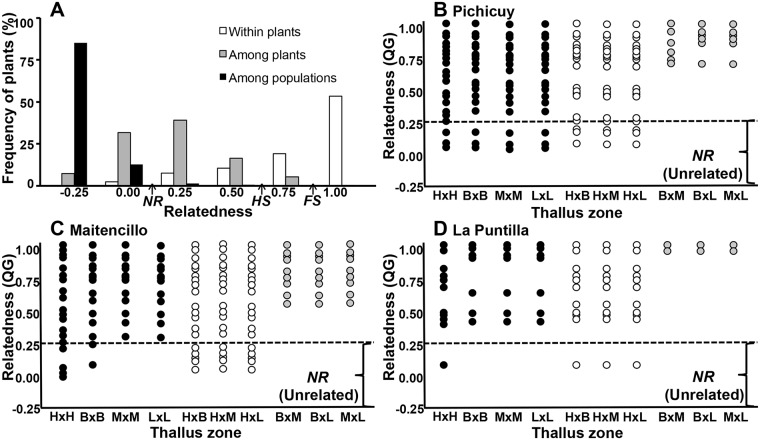
Relatedness among samples. (A) Frequency distribution of the relatedness (QG-value) at three different levels: “within plant” in white, “among plants” in grey, and “among populations” in black. The theoretical values for unrelated individuals (NR), half sibs (HS) and full sibs (FS) are indicated. (B-D) “Within-plant” relatedness (QG- value) comparing different thallus zones in chimeric plants from the three studied populations of *L*. *spicata*. H: Holdfast, B: Basal stipe, M: Medial stipe, L: Lamina. Comparisons among the same thallus zones are showed by black circles; comparisons among holdfast versus axes (stipes and laminas) are showed by grey circles. Comparisons excluding holdfast are represented by white circles.

The relatedness analysis evidenced significant differences between the three levels (F = 4.0; *P<0*.*001*, S2 Table). The “within plant” level had the highest QG value (mean 0.85 ± 0.22) followed by the “among plant” (mean 0.55 ± 0.20) and “among population” levels (mean 0.02 ± 0.20). So, the distribution of frequency showed three peaks with a QG value of 1, 0.25, and -0.25 for the three levels respectively (Fig 3A). However, there were unrelated samples for the three levels. The specific analysis for the “within plant” level showed a high QG value among the different thallus zones from the same plant ranging from 0.52 to 1.0 with significant differences among populations (F_2,42_ = 6.48; P<0.001, Table 2). La Puntilla population had the highest QG value (mean 0.94± 0.08), compared to Maitencillo (mean 0.83± 0.11) and Pichicuy (mean 0.79± 0.15). Therefore, a QG value of less than 0.24 (unrelated genotypes) was found in plants that showed different genotypes, from La Puntilla (LP7), six plants in Maitencillo (MA3, MA5, MA8, MA10, MA11, and MA14) and four plants in Pichicuy (PI4, PI9, PI11, PI12; see asterisk in Tables 1 and 2). “Within-plant” analyses showed that most unrelated genotypes occurred at the holdfast level. We found 6.6% of sampled holdfasts in La Puntilla with unrelated tissues, and 26.6% in the Maitencillo and Pichicuy populations. In contrast, unrelated genotypes were less frequent in the stipes and lamina zones (13% in Maitencillo and 26.6% in Pichicuy) and their axes emerged from unrelated holdfast genotypes (Table 1).

### a) Frequency of IGH and chimerism

Considering the number of different genotypes by plant, populations of *L*. *spicata* showed statistical differences in the frequency of IGH (G = 75.8, df = 2, *P*<0.001), all sampled plants (100%) exhibiting more than one genotype per plant in Pichicuy, 93.3% in Maitencillo, but only 60% (9/15 plants) were found in the La Puntilla population.

In terms of chimerism, using the first approach (plants with genotypes that diverged in 2 or loci), we also found significant differences between the three studied populations (G = 64.8, df = 2, *P* <0.001). The La Puntilla population had the lowest chimerism frequency (33.3%), followed by Maitencillo (66.7%) and Pichicuy (86.7%, Table 3). In the same way, using the second Bayesian approach (more than 2 genetic clusters by plant), we also found significant differences between the three studied populations (G = 57.5, df = 2, *P* <0.001); however, the frequencies of chimerism were lower suggesting a conservative method to estimate chimerism compared to the first approach used in our study. Then La Puntilla population had lowest chimeric frequency (6.7%) followed by Pichicuy (26.7) and Maitencillo (53.3%, Table 3).

**Table 3:** Summary of chimeric and non-chimeric plants (percentages) obtained by the two approaches used in this study, from the three harvested populations of *L*. *spicata*.

	N° of genotypes per plant that diverged in different loci				N° of genetic cluster per plant	
Population	1	≥2 loci	≥3 loci	≥4 loci	1	2
	(Non-chimera)	(Chimera)			(Non-chimera)	(Chimera)
Pichicuy	13.3	86.7	46.7	26.7	73.3	26.7
Maitencillo	33.3	66.7	60.0	40.0	60.0	53.3
La Puntilla	66.7	33.3	20.0	20.0	93.3	6.7

Genetic relatedness analysis in chimeric plants showed that a similar pattern occurs at the “within-plant” level for the three studied *L*. *spicata* populations. Therefore, all populations showed the same pattern of relatedness in the horizontal analysis (HxH, BxB, MxM, LxL; see black dots in Fig 3B and 3D), whereas the vertical analysis showed clonal, high-related and unrelated tissues when comparing holdfasts with the basal, medial and lamina portions of the same stipe (white dots in Fig 3B and 3D). However, relatedness increased when we excluded holdfast tissues from the vertical analysis (see grey dots in Fig 3B and 3D). In all populations therefore, the axes (stipe and lamina) showed the highest relatedness, with the QG value ranging from 0.69–1.00 in Pichicuy, 0.54–1.00 Maitencillo and 0.87–1.00 in La Puntilla.

### b) Genetic diversity at the individual and population levels

**Fig 4:**
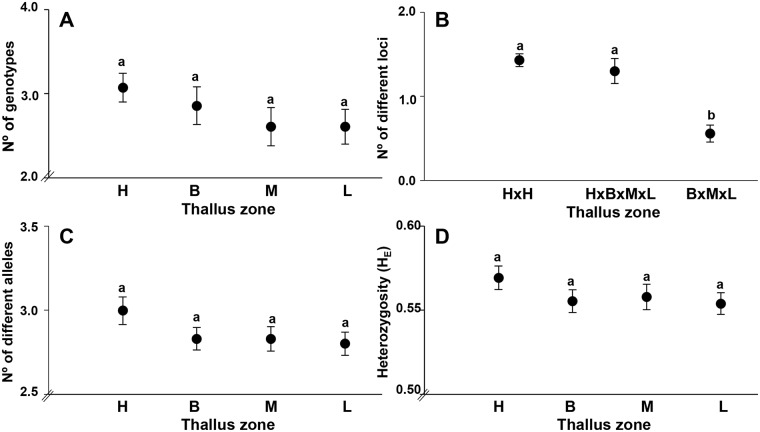
Genetic differences in plants with chimerism comparing different thallus zones. (A) Total number of genotypes. (B) Number of genotypes that differ in 2 or more loci in a paired comparison among different thallus zones; horizontal analysis (HxH) and vertical analysis (HxBxMxL and BxMxL). (C) Number of different alleles. (D) Expected heterozygosity (H_E_). H: Holdfast, B: Basal stipe, M: Medial stipe, L: Lamina. Different superscript letters indicate significant differences (*P <* 0.001).

Chimeric plants showed significant differences between different thallus zones at the “within plant” level. So, we found a higher value of genetic diversity in the holdfast but reduced throughout the apical portion of plants (S1 Dataset, Table 4, Fig 4). Therefore, paired comparison among different thallus zones evidenced that when we included samples from holdfast, the horizontal analysis HxH (mean 1.42 ± 1.2) and vertical analysis HxBxMxL (mean 1.30 ± 1.7) showed highest value of different loci (Fig 4B). In contrast, when we excluded holdfast in the vertical analysis, only comparing stipe and lamina from the same plant (BxMxL) the number of different loci was reduced significantly more than two times (mean 0.55 ± 0.1) (Fig 4B, Wald statistic = 58.3, df = 2, *P*<0.001). A similar tendency of vertical genetic variation “within plant”, though insignificant, was observed in terms of the total number of genotypes (Fig 4A), the number of different alleles (Fig 4C) and expected heterozygosity (H_E_, Fig 4D), where holdfast zones had the highest value compared to stipe and lamina.

**Table 4:** Genetic characterization plants in the different thallus zones in the three harvested populations of *L*. *spicata*. H: Holdfast, B: Basal stipe, M: Medial stipe, L: Lamina.

	N° of different alleles				Expected Heterozygosity (H_E_)			
*Pichicuy*	H	B	M	L	H	B	M	L
PI1	2.66	2.50	2.50	2.50	0.53	0.53	0.53	0.53
PI2	2.50	2.50	3.16	2.50	0.50	0.50	0.47	0.50
PI3	2.50	2.66	2.66	3.00	0.53	0.54	0.54	0.57
PI4	3.66	2.50	2.50	2.50	0.62	0.53	0.53	0.53
PI5	2.83	2.83	2.83	2.83	0.56	0.55	0.57	0.55
PI6	2.50	2.83	2.83	2.83	0.53	0.55	0.57	0.55
PI7	2.50	2.83	2.83	2.50	0.53	0.55	0.57	0.53
PI8	2.50	2.50	2.66	2.50	0.53	0.53	0.54	0.53
PI9	3.66	3.50	2.50	3.33	0.62	0.63	0.53	0.63
PI10	2.50	2.50	2.66	2.66	0.53	0.53	0.55	0.54
PI11	3.00	3.16	3.66	2.66	0.57	0.58	0.65	0.54
PI12	3.66	2.66	3.00	2.50	0.63	0.54	0.59	0.53
PI13	2.83	3.16	2.50	2.50	0.55	0.54	0.53	0.53
PI14	3.16	3.16	3.16	3.16	0.60	0.61	0.61	0.58
PI15	2.66	2.66	2.50	2.50	0.55	0.54	0.53	0.53
***Maitencillo***								
MA1	2.83	2.83	2.50	2.83	0.55	0.55	0.53	0.55
MA2	2.66	2.50	2.50	2.50	0.54	0.53	0.53	0.53
MA3	3.33	2.50	2.66	3.00	0.59	0.53	0.54	0.59
MA4	2.50	2.50	2.83	2.50	0.53	0.53	0.57	0.53
MA5	3.50	2.66	2.50	2.66	0.63	0.54	0.53	0.54
MA6	2.50	2.50	2.50	2.50	0.53	0.53	0.53	0.53
MA7	2.50	2.66	2.66	2.66	0.53	0.55	0.55	0.54
MA8	2.50	2.83	3.50	3.33	0.53	0.55	0.62	0.61
MA9	3.83	3.83	3.33	3.33	0.63	0.63	0.60	0.59
MA10	3.50	2.66	2.83	2.83	0.61	0.54	0.55	0.55
MA11	2.66	3.00	3.83	3.83	0.55	0.57	0.64	0.62
MA12	3.16	3.00	2.83	2.50	0.58	0.57	0.55	0.55
MA13	2.50	2.50	2.66	2.50	0.54	0.54	0.56	0.54
MA14	2.83	3.00	3.16	2.66	0.55	0.57	0.60	0.54
MA15	3.33	3.50	2.50	3.16	0.60	0.65	0.53	0.60
***La Puntilla***								
LP1	2.66	2.66	2.50	2.66	0.54	0.54	0.53	0.54
LP2	2.83	2.66	2.66	2.66	0.55	0.55	0.55	0.54
LP3	2.66	2.66	2.50	2.66	0.55	0.55	0.53	0.55
LP4	2.50	2.50	2.50	2.50	0.53	0.53	0.53	0.53
LP5	3.33	2.66	2.50	2.50	0.59	0.54	0.53	0.53
LP6	2.66	2.50	2.66	3.16	0.54	0.53	0.54	0.58
LP7	3.16	2.33	2.33	2.33	0.58	0.51	0.51	0.49
LP8	2.50	2.50	2.50	2.50	0.53	0.53	0.53	0.53
LP9	2.50	2.66	2.50	2.50	0.53	0.55	0.53	0.53
LP10	2.50	2.50	2.50	2.50	0.53	0.53	0.53	0.53
LP11	2.83	2.66	2.50	2.50	0.55	0.54	0.53	0.53
LP12	2.50	2.50	2.50	2.50	0.53	0.53	0.53	0.53
LP13	2.66	2.50	2.50	2.50	0.54	0.53	0.53	0.53
LP14	2.50	2.50	2.50	2.50	0.53	0.53	0.53	0.53
LP15	2.50	2.50	2.50	2.50	0.53	0.53	0.53	0.53

On the other hand, the three-level AMOVA analysis (Table 5) suggests that genetic variation is mostly explained by differences at the “within-plant” level (39.46%), followed by the “among-population” (34.21%) and “among-plant-within-population” levels (26.32%). This finding supports the previous data that shows different genotypes occurring “within plants” and showed IGH and/or chimerism. Moreover, the F indexes showed statistical differences at all levels. The F_ST_ value, considering all plants, indicated greater differences (F_ST_ = 0.60539, *P*<0.05) compared to the “among-plant-within-population” (F_SC_ = 0.40016, *P*<0.05) and “among-population” (F_CT_ = 0.34214, *P*<0.05) levels.

**Table 5:** Hierarchical AMOVA analyses of spatial scales of differentiation in *L*. *spicata*. All data showed significant differences at *P* <0.001 after 10000 permutations.

Source of variation	df	Sum of squares	Variance components	Percentage of variation
Among populations	2	886654	0.70154	34.21
Among plants within populations	42	940803	0.53977	26.32
Within plants	1755	1420025	0.80913	39.46
Total	1799	3247482	205045	

The re-estimation of genetic diversity considering simultaneously samples from different thallus zones for evaluating the effects of IGH, showed that genetic distances between populations were considerable, suggesting a spatial structuration among the studied populations (Table 6). The fixation index F_ST_ ranged from 0.14–0.28, independent of each thallus zone analyzed (S2 Dataset, Table 6). Theoretically, it has been suggested that values above 0.25 indicated considerable genetic differentiation among populations, meaning in all likelihood that the populations were not breeding with one another at the time [54, 55]. In our study, the population with the lowest frequency of chimeric plants, La Puntilla, had the greatest genetic differentiation, with F_ST_ ranging from 0.20–0.28, compared to the lower genetic differentiation in the Maitencillo and Pichicuy populations, with F_ST_ ranging from 0.14 to 0.15. In all cases, genetic differentiation between populations showed a similar pattern of variation when we compared samples from different thallus zones. Thus, the F_ST_ index increased from the holdfast level to the apical portion of axes (medial stipe and lamina, Table 6).

**Table 6:** Genetic differentiation among populations of *L*. *spicata*. F_ST_ was estimated with 10000 permutations. We considered four different thallus zones simultaneously. All data showed significant differences among populations at *P* <0.001.

	Population		
	Pichicuy	Maitencillo	La Puntilla
**Holdfast samples**			
Pichicuy	-		
Maitencillo	0.146	-	
La Puntilla	0.205	0.266	-
**Basal samples**			
Pichicuy	-		
Maitencillo	0.148	-	
La Puntilla	0.222	0.277	-
**Medial samples**			
Pichicuy	-		
Maitencillo	0.153	-	
La Puntilla	0.230	0.288	-
**Lamina samples**			
Pichicuy	-		
Maitencillo	0.155	-	
La Puntilla	0.231	0.284	-

**Fig 5:**
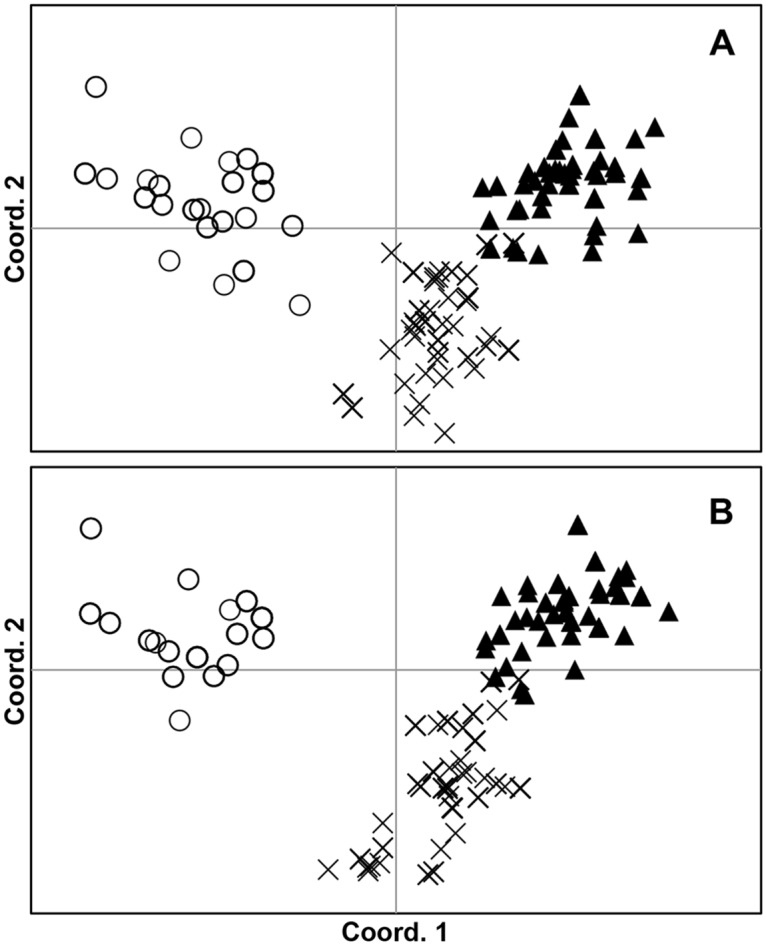
Principal coordinate analyses (PCoA) constructed using genetic covariance matrices among the three studied populations of *L*. *spicata*. Circles represent the La Puntilla population. Triangles correspond to Maitencillo, whereas “X” symbolizes the Pichicuy population. (A) Analysis performed with data from holdfast samples. (B) Analysis performed with data from the lamina samples.

Results of the principal coordinate analyses (PCoA) were consistent with the pattern described above. The first component showed a clear separation of the population in the La Puntilla population, whereas the second component underscored the difference between the Pichicuy and Maitencillo populations (S2 Dataset, Fig 5). The first two principal components explain 55.35% and 56.66% of the distribution, depending on the thallus zone analyzed. Similar to that just described for all genetic data, IGH in different thallus zones affects the genetic differentiation among populations. The close proximity among populations therefore tended to decrease (in some cases overlapping) when we compared the analyses using holdfast samples (Fig 5A) to those using only lamina samples (Fig 5B).

## Discussion

Results found in the three populations of *L*. *spicata*, show a high frequency of chimerism and therefore of individuals with high IGH. The frequency of chimerism was different in each of the two approaches used in this study. Using the first approach (genotypes differences in 2 or more loci per plant), chimerism ranged from 33.3 to 86.7% of the sampled plants, varying according to the studied population. However, considering more than 2 different genetic clusters per plant, chimeric estimation was more conservative, ranging from 6.7 to 53.3% of the sampled plants. In addition, we detected a vertical gradient (from holdfast to lamina) of genetic diversity in chimeric plants, with higher numbers of genotypes, allele divergence and unrelated samples in the holdfasts, which decreased towards the apical portions of the axes (stipes and lamina). Finally, we evidenced that IGH affected genetic differentiation among populations, showed lower levels of F_ST_ index when we compared holdfast than lamina samples.

Regardless of the genetic approaches used, the highest harvested population, Pichicuy showed that 26.7–86.7% of plants were chimeric, followed by Maitencillo (53.3–66.7%) and La Puntilla (6.7–33%). Most of these frequency values fall within the range of values found in the only other study on chimerism frequency for natural populations of this species in Chile *Lessonia spicata* (60–90%, [31]). However, in that study [31] kelp holdfasts were not considered and the occurrence of methodological errors (electrophoresis artifacts) were not evaluated. In our study we found that stipes were genetically homogenous without mixing tissues from base to lamina. Such mixing and chimerism occurred in the holdfast. Therefore, individual stipes and apical lamina only represent a small portion of all the genetic diversity that the holdfast can store. On the other hand, quantification of electrophoretic artifacts decreased the chimerism values originally found in these populations.

Although we found different frequencies of chimerism in the three populations of *Lessonia spicata* studied, we do not know the factors that responsible for these differences. Care was taken to sample populations with similar densities so as to avoid density-dependent differences in chimerism. The three populations also differed in terms of wave exposure. La Puntilla corresponded to a less wave-exposed rocky platform than Pichicuy and Maitencillo. Kelps reduce the rate of fatal kelp wave-induced dislodgements through holdfast aggregation, as evidenced in *Ecklonia radiata* [56]. Coalescent individuals are much more drag resistant than solitary individuals. While aggregation protects plants from dislodgement, it also facilitates chimerism through the fusion of genetically heterogeneous individuals. Thus, any environmental factor that positively selects aggregations (e.g. increased resistance to grazing, increased resistance to wave-induced removal, increased tolerance against desiccation) is likely to increase the frequency of chimerism in a given algal population.

Results also suggest that there may be a positive correlation between chimerism and production. The population from La Puntilla exhibited a low frequency of chimerism as well as the lowest annual weight of harvested biomass. A similar relation appears when comparing populations from Maitencillo with Pichicuy. Even though there may be several factors that influence the harvested-biomass values, these populations were selected because of a similar kelp density, and chimerism might have a role to play here. Studies on red algal chimera have shown positive correlations between survival [21, 23, 24], growth [23, 25], reproduction [21], and environmental stress tolerance [22]. Similarly, in *L*. *spicata* from protected areas, higher reproductive success and reproductive efficiency in the entire thallus has been described for chimeric as opposed to non-chimeric plants [31]. Consequently, in spite of similar plant-density values, kelp populations with a higher number of chimeric plants may have more spores, gametophytes, sporophytes production and greater survival rates, which would also increase the probability of “within plant”, “among plant” and “among population” genetic diversity compared to more homogenous populations. Future studies that incorporate more specific data regarding local conditions such as: habitat (e.g. wave intensity and exposure), and harvesting activities (e.g. access, frequency, and methods used) would allow us to determine whether or not chimerism has major implications for seaweed production.

Data from different thallus zones evidenced that chimerism occurs mostly in holdfasts, showing genetically heterogeneous tissues at this thallus levels, whereas stipes and lamina were more genetically homogeneous, generating a vertical gradient of allele, genotype abundance and divergence. These findings constitute the first report of vertical genetic “within- plant” variation in brown macroalgae and a chimeric pattern similar to the patterns recently described in red algae, but very different from the chimerism described for land plants and animals [25]. On the other hand, since holdfast fusion and chimerism has been shown to have positive ecological implications in terms of an increase in the number of axes [26, 28], reproductive success [31], survival in exposed habitats [29, 56] and resistance against herbivorous effects [29, 30]; in natural populations under exploitation, as is the case of *L*. *spicata*, this type of holdfast could play an important role as a priceless *in-situ* reservoir of natural genetic resources and variability (e.g. revitalizing existing genetic diversity, acting as a reservoir of genetic diversity, and increased resilience in harvested kelp stands compared to non-chimeric holdfasts). This hypothetical role adds to the known ecological role of holdfasts in larval settlement and in providing an area of refuge for invertebrates [57].

Traditionally, studies of genetic diversity have considered the individual, population and regional levels. However, our data showed that the intraorganismal level is another key factor that should be considered. In this way, since kelps are ecosystem bioengineers, and their exploitation along the Chilean coastline has an enormous ecological and socio-economic cost [29, 33, 34, 58, 59], coastal managers need to consider and optimize the genetic diversity of exploited kelp stands at four levels (within-plant, individual, population, and ecosystem) in order to maximize the long-term sustainability of populations they are tasked to protect. Because population viability is sensitive to genetic factors [60], ecosystem resistance and resilience (stability) and the provision of ecosystem services, are often positively correlated with increased biodiversity at the individual and population levels [61]. Therefore, we expect greater “within-plant” genetic diversity due to holdfast fusion and chimerism to increase plant resilience, improve restoration and ecosystem service in terms of habitat provision (e.g. holdfast fusion increase its size, augmenting the habitat for invertebrate recruitment and density) and productivity (e.g. higher number of axes in chimeric plants due to holdfast fusion increases photosynthetic activity) as opposed to genetically homogenous plants. Therefore, future studies and kelp managers should evaluate the significance of chimeric holdfasts as genetic reservoirs for the postharvest recovery of natural populations of brown algae in terms of restoration targets and their contribution to the success of ecosystem restoration.

## Supporting information

S1 Table Allelic and genotype composition in *Lessonia spicata*. Data of allelic and genotype composition of different samples from the three studied populations of *L*. *spicata*. H: Holdfast, B: Basal stipe, M: Medial stipe. L: Lamina.
(DOCX)

S2 Table Samples relatedness in *Lessonia spicata*. Pairwise relatedness summary of different samples at Holdfast, Basal stipe, Medial stipe and Lamina from the three studied populations of *L*. *spicata*. PIC: Pichicuy, MT: Maitencillo, LP: La Puntilla, QGM = Queller and Goodnight (1989) estimator mean.
(XLSX)

S1 Dataset Genetic diversity in *Lessonia spicata*. S3 Table. Raw data formatted to estimate genetic diversity at different thallus zone. PIC: Pichicuy, MT: Maitencillo, LP: La Puntilla. S4 Table Heterozygosity and F-statistics by thallus zone of different samples from the three studied populations of *L*. *spicata*. N: Sample size, Na: Number of alleles, Ne: Number of effective alleles, I: Information Index, Ho: Observed heterozygosity, He: Expected heterozygosity, uHe: Unbiased expected heterozygosity, and F: Fixation index.
(XLSX)

S2 Dataset Genetic distances between populations in *Lessonia spicata*. S5 Table. Raw data formatted to estimate genetic distances using holdfast samples. S6 Table. Pairwise F_ST_ values among populations using holdfast data. S7 Table. Genetic matrix using holdfast data. S1 Text. Principal Coordinates Analysis (PCoA) using holdfast data. Percentage of variation explained by the first 3 axes. S1 Fig. Principal Coordinates (PCoA) using holdfast data from Pichicuy (Pop1), Maitencillo (Pop2), La Puntilla (Pop3). S8 Table. Eigen values by axis and sample eigen vectors using holdfast data. S9 Table. Raw data formatted to estimate genetic distances using basal samples. S10 Table. Pairwise F_ST_ values among populations using basal data. S11 Table. Genetic matrix using basal data. S2 Text. Principal Coordinates Analysis (PCoA) using basal data. Percentage of variation explained by the first 3 axes. S2 Fig. Principal Coordinates (PCoA) using basal data from Pichicuy (Pop1), Maitencillo (Pop2), La Puntilla (Pop3). S12 Table. Eigen values by axis and sample eigen vectors using basal data. S13 Table. Raw data formatted to estimate genetic distances using medial samples. S14 Table. Pairwise F_ST_ values among populations using medial data. S15 Table. Genetic matrix using medial data. S3 Text. Principal Coordinates Analysis (PCoA) using medial data. Percentage of variation explained by the first 3 axes. S13 Fig. Principal Coordinates (PCoA) using medial data from Pichicuy (Pop1), Maitencillo (Pop2), La Puntilla (Pop3). S16 Table. Eigen values by axis and sample eigen vectors using medial data. S17 Table. Raw data formatted to estimate genetic distances using lamina samples. S18 Table. Pairwise F_ST_ values among populations using lamina data. S19 Table. Genetic matrix using lamina data. S4 Text. Principal Coordinates Analysis (PCoA) using lamina data. Percentage of variation explained by the first 3 axes. S4 Fig. Principal Coordinates (PCoA) using lamina data from Pichicuy (Pop1), Maitencillo (Pop2), La Puntilla (Pop3). S20 Table. Eigen values by axis and sample eigen vectors using lamina data.
(XLSX)
