# Supplementary material for: Frequency of chimerism in populations of the kelp Lessonia spicata in central Chile
Source: PLoS One. 2017 Feb 24;12(2):e0169182. doi: 10.1371/journal.pone.0169182 (PMC5325190; doi:10.1371/journal.pone.0169182)
Supplement: S1 Table — Data of allelic and genotype composition of different samples from the three studied populations of L. spicata. H: Holdfast, B: Basal stipe, M: Medial stipe. L: Lamina. (DOCX) [file pone.0169182.s001.docx]

**S1 Table.** **Allelic and genotype composition in *Lessonia spicata***. Data of allelic and genotype composition of different samples from the three studied populations of *L. spicata*. H: Holdfast, B: Basal stipe, M: Medial stipe. L: Lamina

| ♯ | Pop. | Nº of Plant | Replicate | Thallus zone | LESS1T3 | LESS1D4 | LESS1T9 | LESS1T11 | LESS2D22 | LESS2D25 | N° of genotype |
| --- | --- | --- | --- | --- | --- | --- | --- | --- | --- | --- | --- |
| 1 | Pichicuy | PI1 | 1 | H | 136180 | 164166 | 282282 | 155155 | 117117 | 229229 | 1 |
| 2 | Pichicuy | PI1 | 1 | B | 136188 | 164166 | 282282 | 155155 | 117117 | 229229 | 2 |
| 3 | Pichicuy | PI1 | 1 | M | 136188 | 164166 | 282282 | 155155 | 117117 | 229229 | 2 |
| 4 | Pichicuy | PI1 | 1 | L | 136188 | 164166 | 282282 | 155155 | 117117 | 229229 | 2 |
| 5 | Pichicuy | PI1 | 2 | H | 136180 | 164166 | 282282 | 155155 | 117117 | 229229 | 1 |
| 6 | Pichicuy | PI1 | 2 | B | 136180 | 164166 | 282282 | 155155 | 117117 | 229229 | 1 |
| 7 | Pichicuy | PI1 | 2 | M | 136180 | 164166 | 282282 | 155155 | 117117 | 229229 | 1 |
| 8 | Pichicuy | PI1 | 2 | L | 136180 | 164166 | 282282 | 155155 | 117117 | 229229 | 1 |
| 9 | Pichicuy | PI1 | 3 | H | 136180 | 164166 | 282282 | 155155 | 117117 | 229229 | 1 |
| 10 | Pichicuy | PI1 | 3 | B | 136180 | 164166 | 282282 | 155155 | 117117 | 229229 | 1 |
| 11 | Pichicuy | PI1 | 3 | M | 136180 | 164166 | 282282 | 155155 | 117117 | 229229 | 1 |
| 12 | Pichicuy | PI1 | 3 | L | 136180 | 164166 | 282282 | 155155 | 117117 | 229229 | 1 |
| 13 | Pichicuy | PI1 | 4 | H | 136180 | 164166 | 282282 | 155155 | 117117 | 229229 | 1 |
| 14 | Pichicuy | PI1 | 4 | B | 136180 | 164166 | 282282 | 155155 | 117117 | 229229 | 1 |
| 15 | Pichicuy | PI1 | 4 | M | 136180 | 164166 | 282282 | 155155 | 117117 | 229229 | 1 |
| 16 | Pichicuy | PI1 | 4 | L | 136180 | 164166 | 282282 | 155155 | 117117 | 229229 | 1 |
| 17 | Pichicuy | PI1 | 5 | H | 136180 | 164166 | 282282 | 155155 | 117117 | 229229 | 1 |
| 18 | Pichicuy | PI1 | 5 | B | 136180 | 164166 | 282282 | 155155 | 117117 | 229229 | 1 |
| 19 | Pichicuy | PI1 | 5 | M | 136180 | 164166 | 282282 | 155155 | 117117 | 229229 | 1 |
| 20 | Pichicuy | PI1 | 5 | L | 136180 | 164166 | 282282 | 155155 | 117117 | 229229 | 1 |
| 21 | Pichicuy | PI2 | 1 | H | 172172 | 166170 | 284294 | 155155 | 117117 | 229229 | 3 |
| 22 | Pichicuy | PI2 | 1 | B | 172172 | 166170 | 284294 | 155155 | 117117 | 229229 | 3 |
| 23 | Pichicuy | PI2 | 1 | M | 172172 | 166170 | 284294 | 155155 | 117117 | 229229 | 3 |
| 24 | Pichicuy | PI2 | 1 | L | 172172 | 166170 | 284294 | 155155 | 117117 | 229229 | 3 |
| 25 | Pichicuy | PI2 | 2 | H | 172172 | 166170 | 284294 | 155155 | 117117 | 229229 | 3 |
| 26 | Pichicuy | PI2 | 2 | B | 172172 | 166170 | 284294 | 155155 | 117117 | 229229 | 3 |
| 27 | Pichicuy | PI2 | 2 | M | 172172 | 166170 | 284294 | 155155 | 117117 | 229229 | 3 |
| 28 | Pichicuy | PI2 | 2 | L | 172172 | 166170 | 284294 | 155155 | 117117 | 229229 | 3 |
| 29 | Pichicuy | PI2 | 3 | H | 172172 | 166170 | 284294 | 155155 | 117117 | 229229 | 3 |
| 30 | Pichicuy | PI2 | 3 | B | 172172 | 166170 | 284294 | 155155 | 117117 | 229229 | 3 |
| 31 | Pichicuy | PI2 | 3 | M | 172172 | 166170 | 284294 | 155155 | 117117 | 229229 | 3 |
| 32 | Pichicuy | PI2 | 3 | L | 172172 | 166170 | 284294 | 155155 | 117117 | 229229 | 3 |
| 33 | Pichicuy | PI2 | 4 | H | 172172 | 166170 | 284294 | 155155 | 117117 | 229229 | 3 |
| 34 | Pichicuy | PI2 | 4 | B | 172178 | 166166 | 272286 | 155155 | 117117 | 229229 | 4 |
| 35 | Pichicuy | PI2 | 4 | M | 172178 | 166166 | 272286 | 155155 | 117117 | 229229 | 4 |
| 36 | Pichicuy | PI2 | 4 | L | 172178 | 166166 | 272286 | 155155 | 117117 | 229229 | 4 |
| 37 | Pichicuy | PI2 | 5 | H | 172178 | 166166 | 272286 | 155155 | 117117 | 229229 | 4 |
| 38 | Pichicuy | PI2 | 5 | B | 172178 | 166166 | 272286 | 155155 | 117117 | 229229 | 4 |
| 39 | Pichicuy | PI2 | 5 | M | 172178 | 166166 | 272286 | 155155 | 117117 | 229229 | 4 |
| 40 | Pichicuy | PI2 | 5 | L | 172178 | 166166 | 272286 | 155155 | 117117 | 229229 | 4 |
| 41 | Pichicuy | PI3 | 1 | H | 136184 | 166168 | 296296 | 155155 | 117120 | 229229 | 5 |
| 42 | Pichicuy | PI3 | 1 | B | 136184 | 166168 | 296296 | 155155 | 117120 | 229229 | 5 |
| 43 | Pichicuy | PI3 | 1 | M | 136184 | 166168 | 296296 | 155155 | 117120 | 229229 | 5 |
| 44 | Pichicuy | PI3 | 1 | L | 136184 | 166168 | 296296 | 155155 | 117120 | 229229 | 5 |
| 45 | Pichicuy | PI3 | 2 | H | 136184 | 166168 | 296296 | 155155 | 117120 | 229229 | 5 |
| 46 | Pichicuy | PI3 | 2 | B | 136184 | 166168 | 296296 | 155155 | 117117 | 229229 | 6 |
| 47 | Pichicuy | PI3 | 2 | M | 136184 | 166168 | 296296 | 155155 | 117120 | 229229 | 5 |
| 48 | Pichicuy | PI3 | 2 | L | 136184 | 166168 | 296296 | 155155 | 117120 | 229229 | 5 |
| 49 | Pichicuy | PI3 | 3 | H | 136184 | 166168 | 296296 | 155155 | 117120 | 229229 | 5 |
| 50 | Pichicuy | PI3 | 3 | B | 136184 | 166168 | 296296 | 155155 | 117120 | 229229 | 5 |
| 51 | Pichicuy | PI3 | 3 | M | 136184 | 166168 | 296296 | 155155 | 117120 | 229229 | 5 |
| 52 | Pichicuy | PI3 | 3 | L | 136184 | 166168 | 296296 | 155155 | 117117 | 229229 | 6 |
| 53 | Pichicuy | PI3 | 4 | H | 136184 | 166168 | 296296 | 155155 | 117120 | 229229 | 5 |
| 54 | Pichicuy | PI3 | 4 | B | 136184 | 166168 | 296296 | 155155 | 117120 | 229229 | 5 |
| 55 | Pichicuy | PI3 | 4 | M | 136184 | 166168 | 296296 | 155155 | 117120 | 229229 | 5 |
| 56 | Pichicuy | PI3 | 4 | L | 136184 | 166168 | 296296 | 155155 | 117117 | 229229 | 6 |
| 57 | Pichicuy | PI3 | 5 | H | 136184 | 166168 | 296296 | 155155 | 117120 | 229229 | 5 |
| 58 | Pichicuy | PI3 | 5 | B | 136184 | 164164 | 296296 | 155155 | 117120 | 229229 | 7 |
| 59 | Pichicuy | PI3 | 5 | M | 136184 | 166168 | 296296 | 155155 | 117120 | 229229 | 5 |
| 60 | Pichicuy | PI3 | 5 | L | 136184 | 166168 | 296296 | 155155 | 117120 | 229229 | 5 |
| 61 | Pichicuy | PI4 | 1 | H | 172188 | 166166 | 282282 | 155155 | 117117 | 227231 | 8 |
| 62 | Pichicuy | PI4 | 1 | B | 172188 | 166166 | 282282 | 155155 | 117117 | 227231 | 8 |
| 63 | Pichicuy | PI4 | 1 | M | 172188 | 166166 | 282282 | 155155 | 117117 | 227231 | 8 |
| 64 | Pichicuy | PI4 | 1 | L | 172188 | 166166 | 282282 | 155155 | 117117 | 227231 | 8 |
| 65 | Pichicuy | PI4 | 2 | H | 133136 | 162164 | 272286 | 155155 | 117117 | 229229 | 9 |
| 66 | Pichicuy | PI4 | 2 | B | 133136 | 166166 | 272286 | 155155 | 117117 | 229229 | 10 |
| 67 | Pichicuy | PI4 | 2 | M | 133136 | 166166 | 272286 | 155155 | 117117 | 229229 | 10 |
| 68 | Pichicuy | PI4 | 2 | L | 133136 | 166166 | 272286 | 155155 | 117117 | 229229 | 10 |
| 69 | Pichicuy | PI4 | 3 | H | 133136 | 166166 | 272286 | 155155 | 117117 | 229229 | 10 |
| 70 | Pichicuy | PI4 | 3 | B | 133136 | 166166 | 272286 | 155155 | 117117 | 229229 | 10 |
| 71 | Pichicuy | PI4 | 3 | M | 133136 | 166166 | 272286 | 155155 | 117117 | 229229 | 10 |
| 72 | Pichicuy | PI4 | 3 | L | 133136 | 166166 | 272286 | 155155 | 117117 | 229229 | 10 |
| 73 | Pichicuy | PI4 | 4 | H | 133136 | 166166 | 272286 | 155155 | 117117 | 229229 | 10 |
| 74 | Pichicuy | PI4 | 4 | B | 133136 | 166166 | 272286 | 155155 | 117117 | 229229 | 10 |
| 75 | Pichicuy | PI4 | 4 | M | 133136 | 166166 | 272286 | 155155 | 117117 | 229229 | 10 |
| 76 | Pichicuy | PI4 | 4 | L | 133136 | 166166 | 272286 | 155155 | 117117 | 229229 | 10 |
| 77 | Pichicuy | PI4 | 5 | H | 133136 | 166166 | 272286 | 155155 | 117117 | 229229 | 10 |
| 78 | Pichicuy | PI4 | 5 | B | 133136 | 166166 | 272286 | 155155 | 117117 | 229229 | 10 |
| 79 | Pichicuy | PI4 | 5 | M | 133136 | 166166 | 272286 | 155155 | 117117 | 229229 | 10 |
| 80 | Pichicuy | PI4 | 5 | L | 133136 | 166166 | 272286 | 155155 | 117117 | 229229 | 10 |
| 81 | Pichicuy | PI5 | 1 | H | 136192 | 164164 | 282296 | 155155 | 117117 | 229229 | 11 |
| 82 | Pichicuy | PI5 | 1 | B | 136192 | 166166 | 282296 | 155155 | 117117 | 229229 | 12 |
| 83 | Pichicuy | PI5 | 1 | M | 136192 | 166166 | 282296 | 155155 | 117117 | 229229 | 12 |
| 84 | Pichicuy | PI5 | 1 | L | 136192 | 166166 | 282296 | 155155 | 117117 | 229229 | 12 |
| 85 | Pichicuy | PI5 | 2 | H | 136192 | 164166 | 282296 | 155155 | 117117 | 229229 | 13 |
| 86 | Pichicuy | PI5 | 2 | B | 136192 | 166168 | 282296 | 155155 | 117117 | 229229 | 14 |
| 87 | Pichicuy | PI5 | 2 | M | 136192 | 166168 | 282296 | 155155 | 117117 | 229229 | 14 |
| 88 | Pichicuy | PI5 | 2 | L | 136192 | 166168 | 282296 | 155155 | 117117 | 229229 | 14 |
| 89 | Pichicuy | PI5 | 3 | H | 136192 | 164164 | 282296 | 155155 | 117117 | 229229 | 11 |
| 90 | Pichicuy | PI5 | 3 | B | 136192 | 166168 | 282296 | 155155 | 117117 | 229229 | 14 |
| 91 | Pichicuy | PI5 | 3 | M | 136192 | 166168 | 282296 | 155155 | 117117 | 229229 | 14 |
| 92 | Pichicuy | PI5 | 3 | L | 136192 | 166168 | 282296 | 155155 | 117117 | 229229 | 14 |
| 93 | Pichicuy | PI5 | 4 | H | 136192 | 166168 | 282296 | 155155 | 117117 | 229229 | 14 |
| 94 | Pichicuy | PI5 | 4 | B | 136174 | 166168 | 282282 | 155155 | 117117 | 229229 | 15 |
| 95 | Pichicuy | PI5 | 4 | M | 136174 | 166168 | 282282 | 155155 | 117117 | 229229 | 15 |
| 96 | Pichicuy | PI5 | 4 | L | 136174 | 166168 | 282282 | 155155 | 117117 | 229229 | 15 |
| 97 | Pichicuy | PI5 | 5 | H | 136192 | 166168 | 282296 | 155155 | 117117 | 229229 | 14 |
| 98 | Pichicuy | PI5 | 5 | B | 136192 | 166168 | 282296 | 155155 | 117117 | 229229 | 14 |
| 99 | Pichicuy | PI5 | 5 | M | 136192 | 166168 | 282296 | 155155 | 117117 | 229229 | 14 |
| 100 | Pichicuy | PI5 | 5 | L | 136192 | 166168 | 282296 | 155155 | 117117 | 229229 | 14 |

S1 Table. Continuation

| ♯ | Pop. | Nº of Plant | Replicate | Thallus zone | LESS1T3 | LESS1D4 | LESS1T9 | LESS1T11 | LESS2D22 | LESS2D25 | N° of genotype |
| --- | --- | --- | --- | --- | --- | --- | --- | --- | --- | --- | --- |
| 101 | Pichicuy | PI6 | 1 | H | 136180 | 164168 | 282282 | 155155 | 117117 | 229229 | 16 |
| 102 | Pichicuy | PI6 | 1 | B | 136180 | 164168 | 282282 | 155155 | 117117 | 229229 | 16 |
| 103 | Pichicuy | PI6 | 1 | M | 136180 | 164168 | 282282 | 155155 | 117117 | 229229 | 16 |
| 104 | Pichicuy | PI6 | 1 | L | 136180 | 164168 | 282282 | 155155 | 117117 | 229229 | 16 |
| 105 | Pichicuy | PI6 | 2 | H | 136180 | 164168 | 282282 | 155155 | 117117 | 229229 | 16 |
| 106 | Pichicuy | PI6 | 2 | B | 136180 | 164168 | 282282 | 155155 | 117117 | 229229 | 16 |
| 107 | Pichicuy | PI6 | 2 | M | 136180 | 164168 | 282282 | 155155 | 117117 | 229229 | 16 |
| 108 | Pichicuy | PI6 | 2 | L | 136180 | 164168 | 282282 | 155155 | 117117 | 229229 | 16 |
| 109 | Pichicuy | PI6 | 3 | H | 136180 | 164168 | 282282 | 155155 | 117117 | 229229 | 16 |
| 110 | Pichicuy | PI6 | 3 | B | 136192 | 166168 | 282282 | 155155 | 117117 | 229229 | 17 |
| 111 | Pichicuy | PI6 | 3 | M | 136180 | 164168 | 282282 | 155155 | 117117 | 229229 | 16 |
| 112 | Pichicuy | PI6 | 3 | L | 136180 | 164168 | 282282 | 155155 | 117117 | 229229 | 16 |
| 113 | Pichicuy | PI6 | 4 | H | 136136 | 164164 | 282282 | 155155 | 117117 | 229241 | 18 |
| 114 | Pichicuy | PI6 | 4 | B | 136136 | 164164 | 282282 | 155155 | 117117 | 229241 | 18 |
| 115 | Pichicuy | PI6 | 4 | M | 136136 | 164164 | 282282 | 155155 | 117117 | 229241 | 18 |
| 116 | Pichicuy | PI6 | 4 | L | 136136 | 164164 | 282282 | 155155 | 117117 | 229241 | 18 |
| 117 | Pichicuy | PI6 | 5 | H | 136180 | 164168 | 282282 | 155155 | 117117 | 229229 | 16 |
| 118 | Pichicuy | PI6 | 5 | B | 136180 | 164168 | 282282 | 155155 | 117117 | 229229 | 16 |
| 119 | Pichicuy | PI6 | 5 | M | 136180 | 164168 | 282282 | 155155 | 117117 | 229229 | 16 |
| 120 | Pichicuy | PI6 | 5 | L | 136180 | 164168 | 282282 | 155155 | 117117 | 229229 | 16 |
| 121 | Pichicuy | PI7 | 1 | H | 136174 | 166168 | 278278 | 155155 | 117117 | 227229 | 19 |
| 122 | Pichicuy | PI7 | 1 | B | 136174 | 166168 | 278278 | 155155 | 117117 | 227229 | 19 |
| 123 | Pichicuy | PI7 | 1 | M | 136174 | 166168 | 278278 | 155155 | 117117 | 227229 | 19 |
| 124 | Pichicuy | PI7 | 1 | L | 136174 | 166168 | 278278 | 155155 | 117117 | 227229 | 19 |
| 125 | Pichicuy | PI7 | 2 | H | 136174 | 166168 | 278278 | 155155 | 117117 | 227229 | 19 |
| 126 | Pichicuy | PI7 | 2 | B | 136174 | 166168 | 278278 | 155155 | 117117 | 227229 | 19 |
| 127 | Pichicuy | PI7 | 2 | M | 136174 | 166168 | 278278 | 155155 | 117117 | 227229 | 19 |
| 128 | Pichicuy | PI7 | 2 | L | 136174 | 166168 | 278278 | 155155 | 117117 | 227229 | 19 |
| 129 | Pichicuy | PI7 | 3 | H | 136174 | 166168 | 278294 | 155155 | 117117 | 227229 | 20 |
| 130 | Pichicuy | PI7 | 3 | B | 136174 | 166168 | 278282 | 155155 | 117117 | 227229 | 21 |
| 131 | Pichicuy | PI7 | 3 | M | 136174 | 166168 | 278282 | 155155 | 117117 | 227229 | 21 |
| 132 | Pichicuy | PI7 | 3 | L | 136174 | 166168 | 278282 | 155155 | 117117 | 227229 | 21 |
| 133 | Pichicuy | PI7 | 4 | H | 136174 | 166172 | 270282 | 155155 | 117117 | 227229 | 22 |
| 134 | Pichicuy | PI7 | 4 | B | 136174 | 166172 | 270282 | 155155 | 117117 | 227229 | 22 |
| 135 | Pichicuy | PI7 | 4 | M | 136174 | 166172 | 270282 | 155155 | 117117 | 227229 | 22 |
| 136 | Pichicuy | PI7 | 4 | L | 136174 | 166172 | 270282 | 155155 | 117117 | 227229 | 22 |
| 137 | Pichicuy | PI7 | 5 | H | 136174 | 166172 | 270282 | 155155 | 117117 | 227229 | 22 |
| 138 | Pichicuy | PI7 | 5 | B | 136174 | 166172 | 270282 | 155155 | 117117 | 227229 | 22 |
| 139 | Pichicuy | PI7 | 5 | M | 136174 | 166172 | 270282 | 155155 | 117117 | 227229 | 22 |
| 140 | Pichicuy | PI7 | 5 | L | 136174 | 166172 | 270282 | 155155 | 117117 | 227229 | 22 |
| 141 | Pichicuy | PI8 | 1 | H | 136136 | 166166 | 282282 | 155155 | 117117 | 229229 | 23 |
| 142 | Pichicuy | PI8 | 1 | B | 136136 | 166166 | 282282 | 155155 | 117117 | 229229 | 23 |
| 143 | Pichicuy | PI8 | 1 | M | 136136 | 166166 | 282282 | 155155 | 117117 | 229229 | 23 |
| 144 | Pichicuy | PI8 | 1 | L | 136136 | 166166 | 282282 | 155155 | 117117 | 229229 | 23 |
| 145 | Pichicuy | PI8 | 2 | H | 136136 | 166166 | 282282 | 155155 | 117117 | 229229 | 23 |
| 146 | Pichicuy | PI8 | 2 | B | 136136 | 166166 | 282282 | 155155 | 117117 | 229229 | 23 |
| 147 | Pichicuy | PI8 | 2 | M | 136136 | 166166 | 282282 | 155155 | 117117 | 229229 | 23 |
| 148 | Pichicuy | PI8 | 2 | L | 136136 | 166166 | 282282 | 155155 | 117117 | 229229 | 23 |
| 149 | Pichicuy | PI8 | 3 | H | 136136 | 166166 | 282282 | 155155 | 117117 | 229229 | 23 |
| 150 | Pichicuy | PI8 | 3 | B | 136136 | 166166 | 282282 | 155155 | 117117 | 229229 | 23 |
| 151 | Pichicuy | PI8 | 3 | M | 136136 | 166166 | 282282 | 155155 | 117117 | 229229 | 23 |
| 152 | Pichicuy | PI8 | 3 | L | 136136 | 166166 | 282282 | 155155 | 117117 | 229229 | 23 |
| 153 | Pichicuy | PI8 | 4 | H | 136136 | 166166 | 282282 | 155155 | 117117 | 229229 | 23 |
| 154 | Pichicuy | PI8 | 4 | B | 136136 | 164166 | 282282 | 155155 | 117117 | 229229 | 24 |
| 155 | Pichicuy | PI8 | 4 | M | 136136 | 166166 | 282282 | 155155 | 117117 | 229229 | 23 |
| 156 | Pichicuy | PI8 | 4 | L | 136136 | 166166 | 282282 | 155155 | 117117 | 229229 | 23 |
| 157 | Pichicuy | PI8 | 5 | H | 136136 | 166166 | 282282 | 155155 | 117117 | 229229 | 23 |
| 158 | Pichicuy | PI8 | 5 | B | 136136 | 166166 | 282282 | 155155 | 117117 | 229229 | 23 |
| 159 | Pichicuy | PI8 | 5 | M | 136136 | 166166 | 282282 | 155155 | 117117 | 229229 | 23 |
| 160 | Pichicuy | PI8 | 5 | L | 136136 | 166166 | 282282 | 155155 | 117117 | 229229 | 23 |
| 161 | Pichicuy | PI10 | 1 | H | 136200 | 166166 | 278282 | 155155 | 117117 | 229229 | 25 |
| 162 | Pichicuy | PI10 | 1 | B | 133186 | 166166 | 280280 | 155155 | 117117 | 227243 | 26 |
| 163 | Pichicuy | PI10 | 1 | M | 133186 | 166166 | 280280 | 155155 | 117117 | 227229 | 27 |
| 164 | Pichicuy | PI10 | 1 | L | 133186 | 166166 | 280280 | 155155 | 117117 | 227229 | 27 |
| 165 | Pichicuy | PI10 | 2 | H | 136144 | 166166 | 280280 | 155155 | 117117 | 231243 | 28 |
| 166 | Pichicuy | PI10 | 2 | B | 133136 | 166166 | 282282 | 155155 | 117117 | 229229 | 29 |
| 167 | Pichicuy | PI10 | 2 | M | 133136 | 166166 | 272282 | 155155 | 117117 | 229229 | 30 |
| 168 | Pichicuy | PI10 | 2 | L | 133136 | 166166 | 272282 | 155155 | 117117 | 229229 | 30 |
| 169 | Pichicuy | PI10 | 3 | H | 136144 | 166166 | 280280 | 155155 | 117117 | 231243 | 28 |
| 170 | Pichicuy | PI10 | 3 | B | 136144 | 166166 | 280280 | 155155 | 117117 | 231243 | 28 |
| 171 | Pichicuy | PI10 | 3 | M | 136144 | 166166 | 280280 | 155155 | 117117 | 231243 | 28 |
| 172 | Pichicuy | PI10 | 3 | L | 136144 | 166166 | 280280 | 155155 | 117117 | 231243 | 28 |
| 173 | Pichicuy | PI10 | 4 | H | 136144 | 166166 | 280280 | 155155 | 117117 | 231243 | 28 |
| 174 | Pichicuy | PI10 | 4 | B | 136144 | 166166 | 280280 | 155155 | 117117 | 231243 | 28 |
| 175 | Pichicuy | PI10 | 4 | M | 136144 | 166166 | 280280 | 155155 | 117117 | 231243 | 28 |
| 176 | Pichicuy | PI10 | 4 | L | 136144 | 166166 | 280280 | 155155 | 117117 | 231243 | 28 |
| 177 | Pichicuy | PI10 | 5 | H | 136144 | 166166 | 280280 | 155155 | 117117 | 231243 | 28 |
| 178 | Pichicuy | PI10 | 5 | B | 133136 | 166166 | 272284 | 155155 | 117117 | 229229 | 31 |
| 179 | Pichicuy | PI10 | 5 | M | 133136 | 166166 | 272284 | 155155 | 117117 | 229229 | 31 |
| 180 | Pichicuy | PI10 | 5 | L | 133136 | 166166 | 272284 | 155155 | 117117 | 229229 | 31 |
| 181 | Pichicuy | PI11 | 1 | H | 136172 | 164164 | 272272 | 155155 | 117117 | 229229 | 32 |
| 182 | Pichicuy | PI11 | 1 | B | 136172 | 164164 | 272272 | 155155 | 117117 | 229229 | 32 |
| 183 | Pichicuy | PI11 | 1 | M | 136172 | 164164 | 272272 | 155155 | 117117 | 229229 | 32 |
| 184 | Pichicuy | PI11 | 1 | L | 136172 | 164164 | 272272 | 155155 | 117117 | 229229 | 32 |
| 185 | Pichicuy | PI11 | 2 | H | 136172 | 164164 | 272272 | 155155 | 117117 | 229229 | 32 |
| 186 | Pichicuy | PI11 | 2 | B | 136172 | 164164 | 272272 | 155155 | 117117 | 229229 | 32 |
| 187 | Pichicuy | PI11 | 2 | M | 136172 | 164164 | 272272 | 155155 | 117117 | 229229 | 32 |
| 188 | Pichicuy | PI11 | 2 | L | 136172 | 164164 | 272272 | 155155 | 117117 | 229229 | 32 |
| 189 | Pichicuy | PI11 | 3 | H | 136172 | 164164 | 272272 | 155155 | 117117 | 229229 | 32 |
| 190 | Pichicuy | PI11 | 3 | B | 136172 | 164164 | 272272 | 155155 | 117117 | 229229 | 32 |
| 191 | Pichicuy | PI11 | 3 | M | 136172 | 164164 | 272272 | 155155 | 117117 | 229229 | 32 |
| 192 | Pichicuy | PI11 | 3 | L | 136172 | 164164 | 272272 | 155155 | 117117 | 229229 | 32 |
| 193 | Pichicuy | PI11 | 4 | H | 136172 | 164190 | 272272 | 155155 | 117117 | 229229 | 33 |
| 194 | Pichicuy | PI11 | 4 | B | 136172 | 164190 | 272272 | 155155 | 117117 | 229229 | 33 |
| 195 | Pichicuy | PI11 | 4 | M | 136172 | 164164 | 272272 | 155155 | 117117 | 229229 | 32 |
| 196 | Pichicuy | PI11 | 4 | L | 136172 | 164164 | 272272 | 155155 | 117117 | 229229 | 32 |
| 197 | Pichicuy | PI11 | 5 | H | 136172 | 164164 | 272296 | 155155 | 117117 | 229229 | 34 |
| 198 | Pichicuy | PI11 | 5 | B | 136172 | 164164 | 272272 | 155155 | 117117 | 229229 | 32 |
| 199 | Pichicuy | PI11 | 5 | M | 136172 | 164164 | 272272 | 155155 | 117117 | 229229 | 32 |
| 200 | Pichicuy | PI11 | 5 | L | 136172 | 164164 | 272272 | 155155 | 117117 | 229229 | 32 |

S1 Table. Continuation

| ♯ | Pop. | Nº of Plant | Replicate | Thallus zone | LESS1T3 | LESS1D4 | LESS1T9 | LESS1T11 | LESS2D22 | LESS2D25 | N° of genotype |
| --- | --- | --- | --- | --- | --- | --- | --- | --- | --- | --- | --- |
| 201 | Pichicuy | PI12 | 1 | H | 136136 | 166166 | 280286 | 155155 | 117117 | 229229 | 35 |
| 202 | Pichicuy | PI12 | 1 | B | 136136 | 166166 | 280286 | 155155 | 117117 | 229229 | 35 |
| 203 | Pichicuy | PI12 | 1 | M | 136136 | 166166 | 280286 | 155155 | 117117 | 229229 | 35 |
| 204 | Pichicuy | PI12 | 1 | L | 136136 | 166166 | 280286 | 155155 | 117117 | 229229 | 35 |
| 205 | Pichicuy | PI12 | 2 | H | 136172 | 166166 | 280290 | 155155 | 117117 | 231231 | 36 |
| 206 | Pichicuy | PI12 | 2 | B | 172186 | 166166 | 280290 | 155155 | 117117 | 231231 | 37 |
| 207 | Pichicuy | PI12 | 2 | M | 172186 | 166166 | 280290 | 155155 | 117117 | 231231 | 37 |
| 208 | Pichicuy | PI12 | 2 | L | 172186 | 166166 | 280290 | 155155 | 117117 | 231231 | 37 |
| 209 | Pichicuy | PI12 | 3 | H | 136172 | 166166 | 280284 | 155155 | 117117 | 229229 | 38 |
| 210 | Pichicuy | PI12 | 3 | B | 172186 | 166166 | 280290 | 155155 | 117117 | 231231 | 37 |
| 211 | Pichicuy | PI12 | 3 | M | 172186 | 166166 | 280290 | 155155 | 117117 | 231231 | 37 |
| 212 | Pichicuy | PI12 | 3 | L | 172186 | 166166 | 280290 | 155155 | 117117 | 231231 | 37 |
| 213 | Pichicuy | PI12 | 4 | H | 136180 | 166166 | 284296 | 155157 | 117117 | 229229 | 39 |
| 214 | Pichicuy | PI12 | 4 | B | 136180 | 166166 | 286296 | 155157 | 117117 | 229229 | 40 |
| 215 | Pichicuy | PI12 | 4 | M | 136180 | 166166 | 286296 | 155157 | 117117 | 229229 | 40 |
| 216 | Pichicuy | PI12 | 4 | L | 136180 | 166166 | 286296 | 155157 | 117117 | 229229 | 40 |
| 217 | Pichicuy | PI12 | 5 | H | 136180 | 166166 | 284296 | 155157 | 117117 | 229229 | 39 |
| 218 | Pichicuy | PI12 | 5 | B | 136180 | 166166 | 286296 | 155157 | 117117 | 229229 | 40 |
| 219 | Pichicuy | PI12 | 5 | M | 136180 | 166166 | 286296 | 155157 | 117117 | 229229 | 40 |
| 220 | Pichicuy | PI12 | 5 | L | 136180 | 166166 | 286296 | 155157 | 117117 | 229229 | 40 |
| 221 | Pichicuy | PI13 | 1 | H | 136180 | 164166 | 282282 | 155155 | 117117 | 229229 | 1 |
| 222 | Pichicuy | PI13 | 1 | B | 136188 | 164164 | 282282 | 155155 | 117117 | 231231 | 41 |
| 223 | Pichicuy | PI13 | 1 | M | 136188 | 164164 | 282282 | 155155 | 117117 | 231231 | 41 |
| 224 | Pichicuy | PI13 | 1 | L | 136188 | 164164 | 282282 | 155155 | 117117 | 231231 | 41 |
| 225 | Pichicuy | PI13 | 2 | H | 136172 | 166166 | 272272 | 155155 | 117117 | 229243 | 42 |
| 226 | Pichicuy | PI13 | 2 | B | 136172 | 166166 | 272272 | 155155 | 117117 | 229243 | 42 |
| 227 | Pichicuy | PI13 | 2 | M | 136172 | 166166 | 272272 | 155155 | 117117 | 229243 | 42 |
| 228 | Pichicuy | PI13 | 2 | L | 136172 | 166166 | 272272 | 155155 | 117117 | 229243 | 42 |
| 229 | Pichicuy | PI13 | 3 | H | 136136 | 166166 | 272272 | 155155 | 117117 | 229243 | 43 |
| 230 | Pichicuy | PI13 | 3 | B | 136172 | 166166 | 272272 | 155155 | 117117 | 229243 | 42 |
| 231 | Pichicuy | PI13 | 3 | M | 136172 | 166166 | 272272 | 155155 | 117117 | 229243 | 42 |
| 232 | Pichicuy | PI13 | 3 | L | 136172 | 166166 | 272272 | 155155 | 117117 | 229243 | 42 |
| 233 | Pichicuy | PI13 | 4 | H | 136136 | 166166 | 282294 | 155155 | 117117 | 229229 | 44 |
| 234 | Pichicuy | PI13 | 4 | B | 136136 | 166166 | 282294 | 155155 | 117117 | 229229 | 44 |
| 235 | Pichicuy | PI13 | 4 | M | 136136 | 166166 | 282294 | 155155 | 117117 | 229229 | 44 |
| 236 | Pichicuy | PI13 | 4 | L | 136136 | 166166 | 282294 | 155155 | 117117 | 229229 | 44 |
| 237 | Pichicuy | PI13 | 5 | H | 136136 | 166166 | 282294 | 155155 | 117117 | 229229 | 44 |
| 238 | Pichicuy | PI13 | 5 | B | 136136 | 166166 | 282294 | 155155 | 117117 | 229229 | 44 |
| 239 | Pichicuy | PI13 | 5 | M | 136136 | 166166 | 282294 | 155155 | 117117 | 229229 | 44 |
| 240 | Pichicuy | PI13 | 5 | L | 136136 | 166166 | 282294 | 155155 | 117117 | 229229 | 44 |
| 241 | Pichicuy | PI14 | 1 | H | 136136 | 166166 | 280286 | 155155 | 117117 | 227231 | 45 |
| 242 | Pichicuy | PI14 | 1 | B | 136136 | 166166 | 280286 | 155155 | 117117 | 227231 | 45 |
| 243 | Pichicuy | PI14 | 1 | M | 136136 | 166166 | 280286 | 155155 | 117117 | 227231 | 45 |
| 244 | Pichicuy | PI14 | 1 | L | 136136 | 166166 | 280286 | 155155 | 117117 | 227231 | 45 |
| 245 | Pichicuy | PI14 | 2 | H | 136174 | 166166 | 280286 | 155155 | 117117 | 229229 | 46 |
| 246 | Pichicuy | PI14 | 2 | B | 136136 | 166166 | 280286 | 155155 | 117117 | 227231 | 45 |
| 247 | Pichicuy | PI14 | 2 | M | 136136 | 166166 | 280286 | 155155 | 117117 | 227231 | 45 |
| 248 | Pichicuy | PI14 | 2 | L | 136136 | 166166 | 280286 | 155155 | 117117 | 227231 | 45 |
| 249 | Pichicuy | PI14 | 3 | H | 136136 | 166166 | 294294 | 155155 | 117117 | 229229 | 47 |
| 250 | Pichicuy | PI14 | 3 | B | 136136 | 166166 | 286294 | 155155 | 117117 | 229229 | 48 |
| 251 | Pichicuy | PI14 | 3 | M | 136136 | 166166 | 286294 | 155155 | 117117 | 229229 | 48 |
| 252 | Pichicuy | PI14 | 3 | L | 136136 | 166166 | 286294 | 155155 | 117117 | 229229 | 48 |
| 253 | Pichicuy | PI14 | 4 | H | 136136 | 166166 | 286294 | 155155 | 117117 | 229229 | 48 |
| 254 | Pichicuy | PI14 | 4 | B | 136136 | 166166 | 286294 | 155155 | 117117 | 229229 | 48 |
| 255 | Pichicuy | PI14 | 4 | M | 136136 | 166166 | 286294 | 155155 | 117117 | 229229 | 48 |
| 256 | Pichicuy | PI14 | 4 | L | 136136 | 166166 | 286294 | 155155 | 117117 | 229229 | 48 |
| 257 | Pichicuy | PI14 | 5 | H | 136136 | 166166 | 286294 | 155155 | 117117 | 229229 | 48 |
| 258 | Pichicuy | PI14 | 5 | B | 136136 | 166166 | 286294 | 155155 | 117117 | 229229 | 48 |
| 259 | Pichicuy | PI14 | 5 | M | 136136 | 166166 | 286294 | 155155 | 117117 | 229229 | 48 |
| 260 | Pichicuy | PI14 | 5 | L | 136136 | 166166 | 286294 | 155155 | 117117 | 229229 | 48 |
| 261 | Pichicuy | PI15 | 1 | H | 136172 | 166166 | 280280 | 155155 | 117117 | 229245 | 49 |
| 262 | Pichicuy | PI15 | 1 | B | 172184 | 166166 | 272272 | 155155 | 117117 | 229245 | 50 |
| 263 | Pichicuy | PI15 | 1 | M | 172184 | 166166 | 272272 | 155155 | 117117 | 229245 | 50 |
| 264 | Pichicuy | PI15 | 1 | L | 172184 | 166166 | 272272 | 155155 | 117117 | 229245 | 50 |
| 265 | Pichicuy | PI15 | 2 | H | 136172 | 166166 | 272280 | 155155 | 117117 | 229245 | 51 |
| 266 | Pichicuy | PI15 | 2 | B | 172184 | 166166 | 272272 | 155155 | 117117 | 229245 | 50 |
| 267 | Pichicuy | PI15 | 2 | M | 172184 | 166166 | 272272 | 155155 | 117117 | 229245 | 50 |
| 268 | Pichicuy | PI15 | 2 | L | 172184 | 166166 | 272272 | 155155 | 117117 | 229245 | 50 |
| 269 | Pichicuy | PI15 | 3 | H | 136172 | 166166 | 280280 | 155155 | 117117 | 229245 | 49 |
| 270 | Pichicuy | PI15 | 3 | B | 136172 | 166166 | 280280 | 155155 | 117117 | 229245 | 49 |
| 271 | Pichicuy | PI15 | 3 | M | 136172 | 166166 | 280280 | 155155 | 117117 | 229245 | 49 |
| 272 | Pichicuy | PI15 | 3 | L | 136172 | 166166 | 280280 | 155155 | 117117 | 229245 | 49 |
| 273 | Pichicuy | PI15 | 4 | H | 136172 | 166166 | 280280 | 155155 | 117117 | 229245 | 49 |
| 274 | Pichicuy | PI15 | 4 | B | 172184 | 166166 | 272272 | 155155 | 117117 | 229245 | 50 |
| 275 | Pichicuy | PI15 | 4 | M | 172184 | 166166 | 272272 | 155155 | 117117 | 229245 | 50 |
| 276 | Pichicuy | PI15 | 4 | L | 172184 | 166166 | 272272 | 155155 | 117117 | 229245 | 50 |
| 277 | Pichicuy | PI15 | 5 | H | 136172 | 166166 | 280280 | 155155 | 117117 | 229245 | 49 |
| 278 | Pichicuy | PI15 | 5 | B | 136172 | 166166 | 280280 | 155155 | 117117 | 229245 | 49 |
| 279 | Pichicuy | PI15 | 5 | M | 136172 | 166166 | 280280 | 155155 | 117117 | 229245 | 49 |
| 280 | Pichicuy | PI15 | 5 | L | 136172 | 166166 | 280280 | 155155 | 117117 | 229245 | 49 |
| 281 | Pichicuy | PI16 | 1 | H | 136136 | 164164 | 272286 | 155155 | 117117 | 229243 | 52 |
| 282 | Pichicuy | PI16 | 1 | B | 136136 | 164164 | 272286 | 155155 | 117117 | 229243 | 52 |
| 283 | Pichicuy | PI16 | 1 | M | 136136 | 164166 | 272286 | 155155 | 117117 | 229243 | 53 |
| 284 | Pichicuy | PI16 | 1 | L | 136136 | 164166 | 272286 | 155155 | 117117 | 229243 | 53 |
| 285 | Pichicuy | PI16 | 2 | H | 136136 | 164164 | 272286 | 155155 | 117117 | 229243 | 52 |
| 286 | Pichicuy | PI16 | 2 | B | 136136 | 164164 | 272286 | 155155 | 117117 | 229243 | 52 |
| 287 | Pichicuy | PI16 | 2 | M | 136136 | 164164 | 272286 | 155155 | 117117 | 229243 | 52 |
| 288 | Pichicuy | PI16 | 2 | L | 136136 | 164164 | 272286 | 155155 | 117117 | 229243 | 52 |
| 289 | Pichicuy | PI16 | 3 | H | 136136 | 164166 | 272286 | 155155 | 117117 | 229243 | 53 |
| 290 | Pichicuy | PI16 | 3 | B | 136136 | 164164 | 272286 | 155155 | 117117 | 229243 | 52 |
| 291 | Pichicuy | PI16 | 3 | M | 136136 | 164164 | 272286 | 155155 | 117117 | 229243 | 52 |
| 292 | Pichicuy | PI16 | 3 | L | 136136 | 164164 | 272286 | 155155 | 117117 | 229243 | 52 |
| 293 | Pichicuy | PI16 | 4 | H | 136136 | 164164 | 272286 | 155155 | 117117 | 229229 | 54 |
| 294 | Pichicuy | PI16 | 4 | B | 136136 | 164164 | 272286 | 155155 | 117117 | 229243 | 52 |
| 295 | Pichicuy | PI16 | 4 | M | 136136 | 164164 | 272286 | 155155 | 117117 | 229243 | 52 |
| 296 | Pichicuy | PI16 | 4 | L | 136136 | 164164 | 272286 | 155155 | 117117 | 229243 | 52 |
| 297 | Pichicuy | PI16 | 5 | H | 136136 | 164164 | 272286 | 155155 | 117117 | 229243 | 52 |
| 298 | Pichicuy | PI16 | 5 | B | 136136 | 164164 | 272286 | 155155 | 117117 | 229243 | 52 |
| 299 | Pichicuy | PI16 | 5 | M | 136136 | 164164 | 272286 | 155155 | 117117 | 229243 | 52 |
| 300 | Pichicuy | PI16 | 5 | L | 136136 | 164164 | 272286 | 155155 | 117117 | 229243 | 52 |

S1 Table. Continuation

| ♯ | Pop. | Nº of Plant | Replicate | Thallus zone | LESS1T3 | LESS1D4 | LESS1T9 | LESS1T11 | LESS2D22 | LESS2D25 | N° of genotype |
| --- | --- | --- | --- | --- | --- | --- | --- | --- | --- | --- | --- |
| 301 | Maitencillo | MA1 | 1 | H | 136136 | 168174 | 282282 | 155155 | 120122 | 229229 | 55 |
| 302 | Maitencillo | MA1 | 1 | B | 136136 | 168174 | 282282 | 155155 | 120122 | 229229 | 55 |
| 303 | Maitencillo | MA1 | 1 | M | 136136 | 168174 | 282282 | 155155 | 120122 | 229229 | 55 |
| 304 | Maitencillo | MA1 | 1 | L | 136136 | 168174 | 282282 | 155155 | 120122 | 229229 | 55 |
| 305 | Maitencillo | MA1 | 2 | H | 136136 | 166168 | 282282 | 155155 | 120122 | 229229 | 56 |
| 306 | Maitencillo | MA1 | 2 | B | 136136 | 166168 | 282282 | 155155 | 120122 | 229229 | 56 |
| 307 | Maitencillo | MA1 | 2 | M | 136136 | 168174 | 282282 | 155155 | 120122 | 229229 | 55 |
| 308 | Maitencillo | MA1 | 2 | L | 136136 | 168174 | 282282 | 155155 | 120122 | 229229 | 55 |
| 309 | Maitencillo | MA1 | 3 | H | 136136 | 168174 | 282282 | 155155 | 120122 | 229229 | 55 |
| 310 | Maitencillo | MA1 | 3 | B | 136136 | 168174 | 282282 | 155155 | 120122 | 229229 | 55 |
| 311 | Maitencillo | MA1 | 3 | M | 136136 | 168174 | 282282 | 155155 | 120122 | 229229 | 55 |
| 312 | Maitencillo | MA1 | 3 | L | 136136 | 168174 | 282282 | 155155 | 120122 | 229229 | 55 |
| 313 | Maitencillo | MA1 | 4 | H | 136136 | 168174 | 282282 | 155155 | 120122 | 229229 | 55 |
| 314 | Maitencillo | MA1 | 4 | B | 136136 | 168174 | 282282 | 155155 | 120122 | 229229 | 55 |
| 315 | Maitencillo | MA1 | 4 | M | 136136 | 168174 | 282282 | 155155 | 120122 | 229229 | 55 |
| 316 | Maitencillo | MA1 | 4 | L | 136136 | 168174 | 282282 | 155155 | 120122 | 229229 | 55 |
| 317 | Maitencillo | MA1 | 5 | H | 136136 | 164168 | 282282 | 155155 | 120122 | 229229 | 57 |
| 318 | Maitencillo | MA1 | 5 | B | 136136 | 168174 | 282282 | 155155 | 120122 | 229229 | 55 |
| 319 | Maitencillo | MA1 | 5 | M | 136136 | 168174 | 282282 | 155155 | 120122 | 229229 | 55 |
| 320 | Maitencillo | MA1 | 5 | L | 136136 | 168174 | 282282 | 155155 | 120122 | 229229 | 55 |
| 321 | Maitencillo | MA2 | 1 | H | 136136 | 168170 | 290290 | 155155 | 120122 | 229229 | 58 |
| 322 | Maitencillo | MA2 | 1 | B | 136136 | 168170 | 290290 | 155155 | 120122 | 229229 | 58 |
| 323 | Maitencillo | MA2 | 1 | M | 136136 | 168168 | 290290 | 155155 | 120122 | 229229 | 59 |
| 324 | Maitencillo | MA2 | 1 | L | 136136 | 168170 | 290290 | 155155 | 120122 | 229229 | 58 |
| 325 | Maitencillo | MA2 | 2 | H | 136136 | 168170 | 290290 | 155155 | 120122 | 229229 | 58 |
| 326 | Maitencillo | MA2 | 2 | B | 136136 | 168170 | 290290 | 155155 | 120122 | 229229 | 58 |
| 327 | Maitencillo | MA2 | 2 | M | 136136 | 168170 | 290290 | 155155 | 120122 | 229229 | 58 |
| 328 | Maitencillo | MA2 | 2 | L | 136136 | 168170 | 290290 | 155155 | 120122 | 229229 | 58 |
| 329 | Maitencillo | MA2 | 3 | H | 136136 | 168170 | 290290 | 155155 | 120122 | 229229 | 58 |
| 330 | Maitencillo | MA2 | 3 | B | 136136 | 168170 | 290290 | 155155 | 120122 | 229229 | 58 |
| 331 | Maitencillo | MA2 | 3 | M | 136136 | 168170 | 290290 | 155155 | 120122 | 229229 | 58 |
| 332 | Maitencillo | MA2 | 3 | L | 136136 | 168170 | 290290 | 155155 | 120122 | 229229 | 58 |
| 333 | Maitencillo | MA2 | 4 | H | 136136 | 168170 | 290290 | 155155 | 120122 | 229229 | 58 |
| 334 | Maitencillo | MA2 | 4 | B | 136136 | 168170 | 290290 | 155155 | 120122 | 229229 | 58 |
| 335 | Maitencillo | MA2 | 4 | M | 136136 | 168170 | 290290 | 155155 | 120122 | 229229 | 58 |
| 336 | Maitencillo | MA2 | 4 | L | 136136 | 168170 | 290290 | 155155 | 120122 | 229229 | 58 |
| 337 | Maitencillo | MA2 | 5 | H | 136136 | 168170 | 290290 | 155155 | 120122 | 229229 | 58 |
| 338 | Maitencillo | MA2 | 5 | B | 136136 | 168170 | 290290 | 155155 | 120122 | 229229 | 58 |
| 339 | Maitencillo | MA2 | 5 | M | 136136 | 168170 | 290290 | 155155 | 120122 | 229229 | 58 |
| 340 | Maitencillo | MA2 | 5 | L | 136136 | 168170 | 290290 | 155155 | 120122 | 229229 | 58 |
| 341 | Maitencillo | MA3 | 1 | H | 136136 | 166168 | 286292 | 155155 | 120122 | 229229 | 60 |
| 342 | Maitencillo | MA3 | 1 | B | 136172 | 168174 | 272282 | 155155 | 120122 | 229231 | 61 |
| 343 | Maitencillo | MA3 | 1 | M | 136172 | 168174 | 272282 | 155155 | 120122 | 229231 | 61 |
| 344 | Maitencillo | MA3 | 1 | L | 136172 | 168174 | 272282 | 155155 | 120122 | 229231 | 61 |
| 345 | Maitencillo | MA3 | 2 | H | 136172 | 168174 | 272282 | 155155 | 120122 | 229231 | 61 |
| 346 | Maitencillo | MA3 | 2 | B | 136172 | 168174 | 272282 | 155155 | 120122 | 229231 | 61 |
| 347 | Maitencillo | MA3 | 2 | M | 136172 | 168174 | 272282 | 155155 | 120122 | 229231 | 61 |
| 348 | Maitencillo | MA3 | 2 | L | 136172 | 168174 | 272282 | 155155 | 120122 | 229231 | 61 |
| 349 | Maitencillo | MA3 | 3 | H | 136172 | 168174 | 272282 | 155155 | 120122 | 229231 | 61 |
| 350 | Maitencillo | MA3 | 3 | B | 136172 | 168174 | 272282 | 155155 | 120122 | 229231 | 61 |
| 351 | Maitencillo | MA3 | 3 | M | 136172 | 168174 | 272282 | 155155 | 120122 | 229231 | 61 |
| 352 | Maitencillo | MA3 | 3 | L | 136172 | 168174 | 272282 | 155155 | 120122 | 229231 | 61 |
| 353 | Maitencillo | MA3 | 4 | H | 172172 | 168174 | 272282 | 155155 | 120122 | 229231 | 62 |
| 354 | Maitencillo | MA3 | 4 | B | 136172 | 168174 | 272282 | 155155 | 120122 | 229231 | 61 |
| 355 | Maitencillo | MA3 | 4 | M | 136172 | 168174 | 272282 | 155155 | 120122 | 229231 | 61 |
| 356 | Maitencillo | MA3 | 4 | L | 136172 | 168174 | 272282 | 155155 | 120122 | 229231 | 61 |
| 357 | Maitencillo | MA3 | 5 | H | 136172 | 168174 | 272282 | 155155 | 120122 | 229231 | 61 |
| 358 | Maitencillo | MA3 | 5 | B | 136174 | 170174 | 272290 | 155155 | 120122 | 229231 | 63 |
| 359 | Maitencillo | MA3 | 5 | M | 136174 | 170174 | 272290 | 155155 | 120122 | 229231 | 63 |
| 360 | Maitencillo | MA3 | 5 | L | 136174 | 170174 | 272290 | 155155 | 120122 | 229231 | 63 |
| 361 | Maitencillo | MA4 | 1 | H | 136136 | 168174 | 272294 | 155155 | 120122 | 229231 | 64 |
| 362 | Maitencillo | MA4 | 1 | B | 136136 | 168174 | 272294 | 155155 | 120122 | 229231 | 64 |
| 363 | Maitencillo | MA4 | 1 | M | 136136 | 168174 | 272294 | 155155 | 120122 | 229231 | 64 |
| 364 | Maitencillo | MA4 | 1 | L | 136136 | 168174 | 272294 | 155155 | 120122 | 229231 | 64 |
| 365 | Maitencillo | MA4 | 2 | H | 136136 | 168174 | 272294 | 155155 | 120122 | 229231 | 64 |
| 366 | Maitencillo | MA4 | 2 | B | 136136 | 168174 | 272294 | 155155 | 120122 | 229231 | 64 |
| 367 | Maitencillo | MA4 | 2 | M | 136136 | 168174 | 272294 | 155155 | 120122 | 229231 | 64 |
| 368 | Maitencillo | MA4 | 2 | L | 136136 | 168174 | 272294 | 155155 | 120122 | 229231 | 64 |
| 369 | Maitencillo | MA4 | 3 | H | 136136 | 168174 | 272294 | 155155 | 120122 | 229231 | 64 |
| 370 | Maitencillo | MA4 | 3 | B | 136136 | 168174 | 272294 | 155155 | 120122 | 229231 | 64 |
| 371 | Maitencillo | MA4 | 3 | M | 136136 | 168174 | 272294 | 155155 | 120122 | 229231 | 64 |
| 372 | Maitencillo | MA4 | 3 | L | 136136 | 168174 | 272294 | 155155 | 120122 | 229231 | 64 |
| 373 | Maitencillo | MA4 | 4 | H | 136136 | 166168 | 272294 | 155155 | 120122 | 229231 | 65 |
| 374 | Maitencillo | MA4 | 4 | B | 136136 | 166168 | 272294 | 155155 | 120122 | 229231 | 65 |
| 375 | Maitencillo | MA4 | 4 | M | 136136 | 168174 | 272294 | 155155 | 120122 | 229231 | 64 |
| 376 | Maitencillo | MA4 | 4 | L | 136136 | 168174 | 272294 | 155155 | 120122 | 229231 | 64 |
| 377 | Maitencillo | MA4 | 5 | H | 136136 | 168174 | 272294 | 155155 | 120122 | 229231 | 64 |
| 378 | Maitencillo | MA4 | 5 | B | 136136 | 168174 | 272294 | 155155 | 120122 | 229231 | 64 |
| 379 | Maitencillo | MA4 | 5 | M | 136136 | 168174 | 272294 | 155155 | 120122 | 229231 | 64 |
| 380 | Maitencillo | MA4 | 5 | L | 136136 | 168174 | 272294 | 155155 | 120122 | 229231 | 64 |
| 381 | Maitencillo | MA5 | 1 | H | 136172 | 168174 | 282290 | 155155 | 120122 | 229229 | 66 |
| 382 | Maitencillo | MA5 | 1 | B | 136136 | 168168 | 272284 | 155155 | 120122 | 229233 | 67 |
| 383 | Maitencillo | MA5 | 1 | M | 136136 | 168168 | 272284 | 155155 | 120122 | 229233 | 67 |
| 384 | Maitencillo | MA5 | 1 | L | 136136 | 160168 | 272284 | 155155 | 120122 | 229233 | 68 |
| 385 | Maitencillo | MA5 | 2 | H | 172172 | 168174 | 282290 | 155155 | 120122 | 229229 | 69 |
| 386 | Maitencillo | MA5 | 2 | B | 136136 | 168174 | 272284 | 155155 | 120122 | 229233 | 70 |
| 387 | Maitencillo | MA5 | 2 | M | 136136 | 168168 | 272284 | 155155 | 120122 | 229233 | 67 |
| 388 | Maitencillo | MA5 | 2 | L | 136136 | 168168 | 272284 | 155155 | 120122 | 229233 | 67 |
| 389 | Maitencillo | MA5 | 3 | H | 136136 | 168168 | 272284 | 155155 | 120122 | 229233 | 67 |
| 390 | Maitencillo | MA5 | 3 | B | 136136 | 168168 | 272284 | 155155 | 120122 | 229233 | 67 |
| 391 | Maitencillo | MA5 | 3 | M | 136136 | 168168 | 272284 | 155155 | 120122 | 229233 | 67 |
| 392 | Maitencillo | MA5 | 3 | L | 136136 | 168168 | 272284 | 155155 | 120122 | 229233 | 67 |
| 393 | Maitencillo | MA5 | 4 | H | 136136 | 168168 | 272284 | 155155 | 120122 | 229233 | 67 |
| 394 | Maitencillo | MA5 | 4 | B | 136136 | 168168 | 272284 | 155155 | 120122 | 229233 | 67 |
| 395 | Maitencillo | MA5 | 4 | M | 136136 | 168168 | 272284 | 155155 | 120122 | 229233 | 67 |
| 396 | Maitencillo | MA5 | 4 | L | 136136 | 162168 | 272284 | 155155 | 120122 | 229233 | 71 |
| 397 | Maitencillo | MA5 | 5 | H | 136136 | 168168 | 272284 | 155155 | 120122 | 229233 | 67 |
| 398 | Maitencillo | MA5 | 5 | B | 136136 | 168168 | 272284 | 155155 | 120122 | 229233 | 67 |
| 399 | Maitencillo | MA5 | 5 | M | 136136 | 168168 | 272284 | 155155 | 120122 | 229233 | 67 |
| 400 | Maitencillo | MA5 | 5 | L | 136136 | 168168 | 272284 | 155155 | 120122 | 229233 | 67 |

S1 Table. Continuation

| ♯ | Pop. | Nº of Plant | Replicate | Thallus zone | LESS1T3 | LESS1D4 | LESS1T9 | LESS1T11 | LESS2D22 | LESS2D25 | N° of genotype |
| --- | --- | --- | --- | --- | --- | --- | --- | --- | --- | --- | --- |
| 401 | Maitencillo | MA6 | 1 | H | 141174 | 168168 | 282284 | 155155 | 120122 | 229229 | 72 |
| 402 | Maitencillo | MA6 | 1 | B | 141174 | 168168 | 282284 | 155155 | 120122 | 229229 | 72 |
| 403 | Maitencillo | MA6 | 1 | M | 141174 | 168168 | 282284 | 155155 | 120122 | 229229 | 72 |
| 404 | Maitencillo | MA6 | 1 | L | 141174 | 168168 | 282284 | 155155 | 120122 | 229229 | 72 |
| 405 | Maitencillo | MA6 | 2 | H | 141174 | 168168 | 282284 | 155155 | 120122 | 229229 | 72 |
| 406 | Maitencillo | MA6 | 2 | B | 141174 | 168168 | 282284 | 155155 | 120122 | 229229 | 72 |
| 407 | Maitencillo | MA6 | 2 | M | 141174 | 168168 | 282284 | 155155 | 120122 | 229229 | 72 |
| 408 | Maitencillo | MA6 | 2 | L | 141174 | 168168 | 282284 | 155155 | 120122 | 229229 | 72 |
| 409 | Maitencillo | MA6 | 3 | H | 141174 | 168168 | 282284 | 155155 | 120122 | 229229 | 72 |
| 410 | Maitencillo | MA6 | 3 | B | 141174 | 168168 | 282284 | 155155 | 120122 | 229229 | 72 |
| 411 | Maitencillo | MA6 | 3 | M | 141174 | 168168 | 282284 | 155155 | 120122 | 229229 | 72 |
| 412 | Maitencillo | MA6 | 3 | L | 141174 | 168168 | 282284 | 155155 | 120122 | 229229 | 72 |
| 413 | Maitencillo | MA6 | 4 | H | 141174 | 168168 | 282284 | 155155 | 120122 | 229229 | 72 |
| 414 | Maitencillo | MA6 | 4 | B | 141174 | 168168 | 282284 | 155155 | 120122 | 229229 | 72 |
| 415 | Maitencillo | MA6 | 4 | M | 141174 | 168168 | 282284 | 155155 | 120122 | 229229 | 72 |
| 416 | Maitencillo | MA6 | 4 | L | 141174 | 168168 | 282284 | 155155 | 120122 | 229229 | 72 |
| 417 | Maitencillo | MA6 | 5 | H | 141174 | 168168 | 282284 | 155155 | 120122 | 229229 | 72 |
| 418 | Maitencillo | MA6 | 5 | B | 141174 | 168168 | 282284 | 155155 | 120122 | 229229 | 72 |
| 419 | Maitencillo | MA6 | 5 | M | 141174 | 168168 | 282284 | 155155 | 120122 | 229229 | 72 |
| 420 | Maitencillo | MA6 | 5 | L | 141174 | 168168 | 282284 | 155155 | 120122 | 229229 | 72 |
| 421 | Maitencillo | MA7 | 1 | H | 136172 | 156168 | 284284 | 155155 | 120122 | 229237 | 73 |
| 422 | Maitencillo | MA7 | 1 | B | 136172 | 156168 | 284284 | 155155 | 120122 | 229237 | 73 |
| 423 | Maitencillo | MA7 | 1 | M | 136172 | 156168 | 284284 | 155155 | 120122 | 229237 | 73 |
| 424 | Maitencillo | MA7 | 1 | L | 136172 | 156168 | 284284 | 155155 | 120122 | 229237 | 73 |
| 425 | Maitencillo | MA7 | 2 | H | 136172 | 156168 | 284284 | 155155 | 120122 | 229237 | 73 |
| 426 | Maitencillo | MA7 | 2 | B | 136172 | 156168 | 284284 | 155155 | 120122 | 229237 | 73 |
| 427 | Maitencillo | MA7 | 2 | M | 136172 | 156168 | 284284 | 155155 | 120122 | 229237 | 73 |
| 428 | Maitencillo | MA7 | 2 | L | 136172 | 156168 | 284284 | 155155 | 120122 | 229237 | 73 |
| 429 | Maitencillo | MA7 | 3 | H | 136172 | 156168 | 284292 | 155155 | 120122 | 229237 | 74 |
| 430 | Maitencillo | MA7 | 3 | B | 136172 | 156168 | 284292 | 155155 | 120122 | 229237 | 74 |
| 431 | Maitencillo | MA7 | 3 | M | 136172 | 156168 | 284292 | 155155 | 120122 | 229237 | 74 |
| 432 | Maitencillo | MA7 | 3 | L | 136172 | 156168 | 284292 | 155155 | 120122 | 229237 | 74 |
| 433 | Maitencillo | MA7 | 4 | H | 136172 | 156168 | 284284 | 155155 | 120122 | 229237 | 73 |
| 434 | Maitencillo | MA7 | 4 | B | 136172 | 156168 | 284284 | 155155 | 120122 | 229237 | 73 |
| 435 | Maitencillo | MA7 | 4 | M | 136172 | 156168 | 284284 | 155155 | 120122 | 229237 | 73 |
| 436 | Maitencillo | MA7 | 4 | L | 136172 | 156168 | 284284 | 155155 | 120122 | 229237 | 73 |
| 437 | Maitencillo | MA7 | 5 | H | 136172 | 156168 | 284292 | 155155 | 120122 | 229237 | 74 |
| 438 | Maitencillo | MA7 | 5 | B | 136172 | 156168 | 284292 | 155155 | 120122 | 229237 | 74 |
| 439 | Maitencillo | MA7 | 5 | M | 136172 | 156168 | 284292 | 155155 | 120122 | 229237 | 74 |
| 440 | Maitencillo | MA7 | 5 | L | 136172 | 156168 | 284292 | 155155 | 120122 | 229237 | 74 |
| 441 | Maitencillo | MA8 | 1 | H | 136174 | 168174 | 282284 | 155155 | 120120 | 229229 | 75 |
| 442 | Maitencillo | MA8 | 1 | B | 136174 | 168174 | 282284 | 155155 | 120120 | 229229 | 75 |
| 443 | Maitencillo | MA8 | 1 | M | 136174 | 168174 | 282284 | 155155 | 120120 | 229229 | 75 |
| 444 | Maitencillo | MA8 | 1 | L | 136174 | 168174 | 282284 | 155155 | 120120 | 229229 | 75 |
| 445 | Maitencillo | MA8 | 2 | H | 136174 | 168174 | 282284 | 155155 | 120120 | 229229 | 75 |
| 446 | Maitencillo | MA8 | 2 | B | 136174 | 168174 | 282284 | 155155 | 120120 | 229229 | 75 |
| 447 | Maitencillo | MA8 | 2 | M | 136174 | 168174 | 282284 | 155155 | 120120 | 229229 | 75 |
| 448 | Maitencillo | MA8 | 2 | L | 136174 | 168174 | 282284 | 155155 | 120120 | 229229 | 75 |
| 449 | Maitencillo | MA8 | 3 | H | 136174 | 168174 | 282284 | 155155 | 120120 | 229229 | 75 |
| 450 | Maitencillo | MA8 | 3 | B | 136136 | 168174 | 282282 | 155155 | 120120 | 229231 | 76 |
| 451 | Maitencillo | MA8 | 3 | M | 136136 | 168168 | 282282 | 155155 | 120120 | 229231 | 77 |
| 452 | Maitencillo | MA8 | 3 | L | 136136 | 168174 | 282282 | 155155 | 120120 | 229231 | 76 |
| 453 | Maitencillo | MA8 | 4 | H | 136174 | 168174 | 282284 | 155155 | 120120 | 229229 | 75 |
| 454 | Maitencillo | MA8 | 4 | B | 136174 | 168174 | 282284 | 155155 | 120122 | 229229 | 78 |
| 455 | Maitencillo | MA8 | 4 | M | 136174 | 168174 | 282284 | 155155 | 110110 | 229229 | 79 |
| 456 | Maitencillo | MA8 | 4 | L | 136174 | 168174 | 282284 | 155155 | 120120 | 229229 | 75 |
| 457 | Maitencillo | MA8 | 5 | H | 136174 | 149160 | 282284 | 155155 | 110110 | 229229 | 80 |
| 458 | Maitencillo | MA8 | 5 | B | 136174 | 149164 | 282284 | 155155 | 110110 | 229229 | 81 |
| 459 | Maitencillo | MA8 | 5 | M | 136174 | 168174 | 282284 | 155155 | 120120 | 229229 | 75 |
| 460 | Maitencillo | MA8 | 5 | L | 136174 | 168174 | 282284 | 155155 | 120120 | 229229 | 75 |
| 461 | Maitencillo | MA9 | 1 | H | 136174 | 168168 | 282282 | 155155 | 110110 | 229229 | 82 |
| 462 | Maitencillo | MA9 | 1 | B | 136174 | 160168 | 282282 | 155155 | 120120 | 229229 | 83 |
| 463 | Maitencillo | MA9 | 1 | M | 136174 | 166168 | 282282 | 155155 | 120120 | 229229 | 84 |
| 464 | Maitencillo | MA9 | 1 | L | 136172 | 168172 | 282282 | 155155 | 120120 | 229229 | 85 |
| 465 | Maitencillo | MA9 | 2 | H | 136172 | 168174 | 282282 | 155155 | 120122 | 229229 | 86 |
| 466 | Maitencillo | MA9 | 2 | B | 136172 | 166168 | 282282 | 155155 | 120122 | 229229 | 87 |
| 467 | Maitencillo | MA9 | 2 | M | 174174 | 166168 | 282282 | 155155 | 120120 | 229229 | 88 |
| 468 | Maitencillo | MA9 | 2 | L | 136172 | 168174 | 282282 | 155155 | 120122 | 229229 | 86 |
| 469 | Maitencillo | MA9 | 3 | H | 136172 | 158168 | 282282 | 155155 | 114114 | 229229 | 89 |
| 470 | Maitencillo | MA9 | 3 | B | 136172 | 158168 | 282282 | 155155 | 120120 | 229229 | 90 |
| 471 | Maitencillo | MA9 | 3 | M | 136172 | 168168 | 282282 | 155155 | 120120 | 229229 | 91 |
| 472 | Maitencillo | MA9 | 3 | L | 136172 | 168168 | 282282 | 155155 | 110110 | 229229 | 92 |
| 473 | Maitencillo | MA9 | 4 | H | 136172 | 168168 | 282282 | 155155 | 120120 | 229229 | 91 |
| 474 | Maitencillo | MA9 | 4 | B | 172172 | 160168 | 282282 | 155155 | 120120 | 229229 | 93 |
| 475 | Maitencillo | MA9 | 4 | M | 136172 | 160164 | 282282 | 155155 | 120120 | 229229 | 94 |
| 476 | Maitencillo | MA9 | 4 | L | 136172 | 168168 | 282282 | 155155 | 120122 | 229229 | 95 |
| 477 | Maitencillo | MA9 | 5 | H | 136172 | 166168 | 282282 | 155155 | 110110 | 229229 | 96 |
| 478 | Maitencillo | MA9 | 5 | B | 136172 | 166168 | 282282 | 155155 | 120120 | 229229 | 97 |
| 479 | Maitencillo | MA9 | 5 | M | 136172 | 160168 | 282282 | 155155 | 120120 | 229229 | 98 |
| 480 | Maitencillo | MA9 | 5 | L | 136172 | 166168 | 282282 | 155155 | 120120 | 229229 | 97 |
| 481 | Maitencillo | MA10 | 1 | H | 136172 | 168174 | 282282 | 155155 | 120122 | 229231 | 99 |
| 482 | Maitencillo | MA10 | 1 | B | 172172 | 168174 | 282282 | 155155 | 122122 | 229231 | 100 |
| 483 | Maitencillo | MA10 | 1 | M | 136172 | 168174 | 282284 | 155155 | 120122 | 229231 | 101 |
| 484 | Maitencillo | MA10 | 1 | L | 136172 | 168174 | 282284 | 155155 | 120122 | 229231 | 101 |
| 485 | Maitencillo | MA10 | 2 | H | 136172 | 168174 | 249249 | 155155 | 122128 | 229231 | 102 |
| 486 | Maitencillo | MA10 | 2 | B | 136172 | 168174 | 282284 | 155155 | 122122 | 229231 | 103 |
| 487 | Maitencillo | MA10 | 2 | M | 136172 | 168174 | 282284 | 155155 | 120122 | 229231 | 101 |
| 488 | Maitencillo | MA10 | 2 | L | 136172 | 168174 | 282284 | 155155 | 120122 | 229231 | 101 |
| 489 | Maitencillo | MA10 | 3 | H | 136172 | 168174 | 282284 | 155155 | 120122 | 229231 | 101 |
| 490 | Maitencillo | MA10 | 3 | B | 136172 | 168174 | 282284 | 155155 | 120122 | 229231 | 101 |
| 491 | Maitencillo | MA10 | 3 | M | 136172 | 168174 | 282284 | 155155 | 120122 | 229231 | 101 |
| 492 | Maitencillo | MA10 | 3 | L | 136172 | 168174 | 282284 | 155155 | 120122 | 229231 | 101 |
| 493 | Maitencillo | MA10 | 4 | H | 136172 | 182187 | 282284 | 155155 | 120122 | 229231 | 104 |
| 494 | Maitencillo | MA10 | 4 | B | 136172 | 168174 | 282284 | 155155 | 120122 | 229231 | 101 |
| 495 | Maitencillo | MA10 | 4 | M | 136172 | 168174 | 282284 | 155155 | 120122 | 229231 | 101 |
| 496 | Maitencillo | MA10 | 4 | L | 136172 | 168174 | 282284 | 155155 | 120122 | 229231 | 101 |
| 497 | Maitencillo | MA10 | 5 | H | 136172 | 168174 | 282284 | 155155 | 120122 | 229231 | 101 |
| 498 | Maitencillo | MA10 | 5 | B | 136172 | 168174 | 282284 | 155155 | 120122 | 229231 | 101 |
| 499 | Maitencillo | MA10 | 5 | M | 136136 | 168174 | 282282 | 155155 | 120122 | 229231 | 105 |
| 500 | Maitencillo | MA10 | 5 | L | 136172 | 168174 | 282284 | 155155 | 120122 | 229231 | 101 |

S1 Table. Continuation

| ♯ | Pop. | Nº of Plant | Replicate | Thallus zone | LESS1T3 | LESS1D4 | LESS1T9 | LESS1T11 | LESS2D22 | LESS2D25 | N° of genotype |
| --- | --- | --- | --- | --- | --- | --- | --- | --- | --- | --- | --- |
| 501 | Maitencillo | MA11 | 1 | H | 136174 | 168174 | 280282 | 155155 | 120120 | 229231 | 106 |
| 502 | Maitencillo | MA11 | 1 | B | 136174 | 168174 | 282282 | 155155 | 120120 | 229231 | 107 |
| 503 | Maitencillo | MA11 | 1 | M | 136174 | 168174 | 282282 | 155155 | 120120 | 229231 | 107 |
| 504 | Maitencillo | MA11 | 1 | L | 136174 | 168174 | 280282 | 155155 | 120120 | 229231 | 106 |
| 505 | Maitencillo | MA11 | 2 | H | 136174 | 168174 | 280282 | 155155 | 120120 | 229231 | 106 |
| 506 | Maitencillo | MA11 | 2 | B | 136174 | 168174 | 280282 | 155155 | 120120 | 229231 | 106 |
| 507 | Maitencillo | MA11 | 2 | M | 136174 | 168174 | 280282 | 155155 | 120122 | 229231 | 108 |
| 508 | Maitencillo | MA11 | 2 | L | 136174 | 168174 | 280290 | 155155 | 117120 | 229231 | 109 |
| 509 | Maitencillo | MA11 | 3 | H | 136174 | 168174 | 280282 | 155155 | 117120 | 229231 | 110 |
| 510 | Maitencillo | MA11 | 3 | B | 136174 | 168174 | 280282 | 155155 | 117120 | 229231 | 110 |
| 511 | Maitencillo | MA11 | 3 | M | 136174 | 156156 | 280282 | 155155 | 120120 | 229231 | 111 |
| 512 | Maitencillo | MA11 | 3 | L | 136174 | 168174 | 280282 | 155155 | 120125 | 229231 | 112 |
| 513 | Maitencillo | MA11 | 4 | H | 136174 | 168174 | 282290 | 155155 | 117120 | 229231 | 113 |
| 514 | Maitencillo | MA11 | 4 | B | 136174 | 168174 | 282282 | 155155 | 117120 | 229231 | 114 |
| 515 | Maitencillo | MA11 | 4 | M | 136174 | 164166 | 282282 | 155155 | 117120 | 229231 | 115 |
| 516 | Maitencillo | MA11 | 4 | L | 136174 | 185189 | 282290 | 155155 | 117120 | 229231 | 116 |
| 517 | Maitencillo | MA11 | 5 | H | 136174 | 168174 | 282290 | 155155 | 120125 | 229231 | 117 |
| 518 | Maitencillo | MA11 | 5 | B | 136174 | 164168 | 282290 | 155155 | 120125 | 229231 | 118 |
| 519 | Maitencillo | MA11 | 5 | M | 136174 | 160160 | 282290 | 155155 | 120120 | 229231 | 119 |
| 520 | Maitencillo | MA11 | 5 | L | 136174 | 168174 | 282290 | 155155 | 120120 | 229231 | 120 |
| 521 | Maitencillo | MA12 | 1 | H | 136174 | 168174 | 282282 | 155155 | 117120 | 229231 | 114 |
| 522 | Maitencillo | MA12 | 1 | B | 136174 | 168168 | 282282 | 155155 | 117120 | 229231 | 121 |
| 523 | Maitencillo | MA12 | 1 | M | 136174 | 166168 | 282282 | 155155 | 117120 | 229231 | 122 |
| 524 | Maitencillo | MA12 | 1 | L | 136136 | 160168 | 282282 | 155155 | 117120 | 229231 | 123 |
| 525 | Maitencillo | MA12 | 2 | H | 136174 | 166168 | 282282 | 155155 | 117120 | 229231 | 122 |
| 526 | Maitencillo | MA12 | 2 | B | 136174 | 149168 | 282290 | 155155 | 117120 | 229231 | 124 |
| 527 | Maitencillo | MA12 | 2 | M | 136174 | 168168 | 282290 | 155155 | 117120 | 229231 | 125 |
| 528 | Maitencillo | MA12 | 2 | L | 136174 | 168168 | 282282 | 155155 | 117120 | 229231 | 121 |
| 529 | Maitencillo | MA12 | 3 | H | 136174 | 168168 | 282290 | 155155 | 117120 | 229231 | 125 |
| 530 | Maitencillo | MA12 | 3 | B | 136174 | 168179 | 282290 | 155155 | 117120 | 229231 | 126 |
| 531 | Maitencillo | MA12 | 3 | M | 136174 | 168168 | 282290 | 155155 | 117120 | 229231 | 125 |
| 532 | Maitencillo | MA12 | 3 | L | 136174 | 168168 | 282290 | 155155 | 117120 | 229231 | 125 |
| 533 | Maitencillo | MA12 | 4 | H | 136174 | 168168 | 282290 | 155155 | 117120 | 229231 | 125 |
| 534 | Maitencillo | MA12 | 4 | B | 136174 | 158168 | 282290 | 155155 | 117120 | 229231 | 127 |
| 535 | Maitencillo | MA12 | 4 | M | 136174 | 164168 | 282290 | 155155 | 117120 | 229231 | 128 |
| 536 | Maitencillo | MA12 | 4 | L | 136174 | 166168 | 282290 | 155155 | 117120 | 229231 | 129 |
| 537 | Maitencillo | MA12 | 5 | H | 136174 | 166168 | 282290 | 155155 | 117120 | 229231 | 129 |
| 538 | Maitencillo | MA12 | 5 | B | 136174 | 166168 | 282290 | 155155 | 117120 | 229231 | 129 |
| 539 | Maitencillo | MA12 | 5 | M | 136174 | 166168 | 282290 | 155155 | 117120 | 229231 | 129 |
| 540 | Maitencillo | MA12 | 5 | L | 136174 | 166168 | 282290 | 155155 | 117120 | 229231 | 129 |
| 541 | Maitencillo | MA13 | 1 | H | 132136 | 168174 | 282290 | 155155 | 117120 | 229231 | 130 |
| 542 | Maitencillo | MA13 | 1 | B | 132172 | 168174 | 282290 | 155155 | 117120 | 229231 | 131 |
| 543 | Maitencillo | MA13 | 1 | M | 132172 | 168174 | 282290 | 155155 | 117120 | 229231 | 131 |
| 544 | Maitencillo | MA13 | 1 | L | 132172 | 168174 | 282290 | 155155 | 117120 | 229231 | 131 |
| 545 | Maitencillo | MA13 | 2 | H | 132172 | 168174 | 282290 | 155155 | 117120 | 229231 | 131 |
| 546 | Maitencillo | MA13 | 2 | B | 132172 | 168174 | 282290 | 155155 | 117120 | 229231 | 131 |
| 547 | Maitencillo | MA13 | 2 | M | 132172 | 168174 | 282290 | 155155 | 117120 | 229231 | 131 |
| 548 | Maitencillo | MA13 | 2 | L | 132172 | 168174 | 282290 | 155155 | 117120 | 229231 | 131 |
| 549 | Maitencillo | MA13 | 3 | H | 132172 | 168174 | 286290 | 155155 | 117120 | 229231 | 132 |
| 550 | Maitencillo | MA13 | 3 | B | 132172 | 168174 | 286290 | 155155 | 117120 | 229231 | 132 |
| 551 | Maitencillo | MA13 | 3 | M | 132172 | 168174 | 286290 | 155155 | 117120 | 229231 | 132 |
| 552 | Maitencillo | MA13 | 3 | L | 132172 | 168174 | 286290 | 155155 | 117120 | 229231 | 132 |
| 553 | Maitencillo | MA13 | 4 | H | 132136 | 168174 | 282290 | 155155 | 117120 | 229231 | 130 |
| 554 | Maitencillo | MA13 | 4 | B | 132136 | 168174 | 282290 | 155155 | 117120 | 229231 | 130 |
| 555 | Maitencillo | MA13 | 4 | M | 132136 | 168174 | 282290 | 155155 | 117120 | 229231 | 130 |
| 556 | Maitencillo | MA13 | 4 | L | 132136 | 168174 | 282290 | 155155 | 117120 | 229231 | 130 |
| 557 | Maitencillo | MA13 | 5 | H | 132172 | 168174 | 282290 | 155155 | 117120 | 229231 | 131 |
| 558 | Maitencillo | MA13 | 5 | B | 132172 | 168174 | 282290 | 155155 | 117120 | 229231 | 131 |
| 559 | Maitencillo | MA13 | 5 | M | 132172 | 168174 | 282290 | 155155 | 117120 | 229231 | 131 |
| 560 | Maitencillo | MA13 | 5 | L | 132172 | 168174 | 282290 | 155155 | 117120 | 229231 | 131 |
| 561 | Maitencillo | MA14 | 1 | H | 136174 | 166168 | 282284 | 155155 | 117120 | 229231 | 133 |
| 562 | Maitencillo | MA14 | 1 | B | 136174 | 166168 | 282284 | 155157 | 117120 | 229231 | 134 |
| 563 | Maitencillo | MA14 | 1 | M | 136174 | 166168 | 282284 | 155155 | 117120 | 229231 | 133 |
| 564 | Maitencillo | MA14 | 1 | L | 136174 | 166168 | 282284 | 155155 | 117120 | 229231 | 133 |
| 565 | Maitencillo | MA14 | 2 | H | 136174 | 166168 | 282290 | 155155 | 117120 | 229231 | 129 |
| 566 | Maitencillo | MA14 | 2 | B | 136174 | 168168 | 280282 | 155155 | 117120 | 229229 | 135 |
| 567 | Maitencillo | MA14 | 2 | M | 136136 | 168168 | 280282 | 155155 | 117120 | 229229 | 136 |
| 568 | Maitencillo | MA14 | 2 | L | 136174 | 168168 | 280282 | 155157 | 117120 | 229229 | 137 |
| 569 | Maitencillo | MA14 | 3 | H | 136174 | 166168 | 280282 | 155155 | 117120 | 229229 | 138 |
| 570 | Maitencillo | MA14 | 3 | B | 136174 | 166168 | 280282 | 155155 | 117120 | 229229 | 138 |
| 571 | Maitencillo | MA14 | 3 | M | 136174 | 166168 | 280282 | 155155 | 117120 | 229229 | 138 |
| 572 | Maitencillo | MA14 | 3 | L | 136174 | 166168 | 280282 | 155155 | 117120 | 229229 | 138 |
| 573 | Maitencillo | MA14 | 4 | H | 136174 | 168170 | 280282 | 155155 | 120125 | 229229 | 139 |
| 574 | Maitencillo | MA14 | 4 | B | 136174 | 166168 | 280282 | 155155 | 120125 | 229229 | 140 |
| 575 | Maitencillo | MA14 | 4 | M | 136174 | 168168 | 280282 | 155155 | 120125 | 229229 | 141 |
| 576 | Maitencillo | MA14 | 4 | L | 136174 | 168168 | 280282 | 155155 | 117120 | 229229 | 135 |
| 577 | Maitencillo | MA14 | 5 | H | 136174 | 166168 | 280282 | 155155 | 117120 | 229229 | 138 |
| 578 | Maitencillo | MA14 | 5 | B | 136174 | 166168 | 280282 | 155155 | 117120 | 229229 | 138 |
| 579 | Maitencillo | MA14 | 5 | M | 136174 | 166168 | 280282 | 155155 | 117120 | 229229 | 138 |
| 580 | Maitencillo | MA14 | 5 | L | 136174 | 166168 | 280282 | 155155 | 117120 | 229229 | 138 |
| 581 | Maitencillo | MA15 | 1 | H | 136172 | 168174 | 280294 | 155155 | 120122 | 229229 | 142 |
| 582 | Maitencillo | MA15 | 1 | B | 136172 | 166168 | 282290 | 155155 | 120122 | 229229 | 143 |
| 583 | Maitencillo | MA15 | 1 | M | 136172 | 166168 | 282290 | 155155 | 120122 | 229229 | 143 |
| 584 | Maitencillo | MA15 | 1 | L | 136172 | 160168 | 282290 | 155155 | 120122 | 229229 | 144 |
| 585 | Maitencillo | MA15 | 2 | H | 136172 | 168174 | 282290 | 155155 | 120122 | 229229 | 66 |
| 586 | Maitencillo | MA15 | 2 | B | 136172 | 156168 | 282290 | 155155 | 118120 | 229229 | 145 |
| 587 | Maitencillo | MA15 | 2 | M | 136172 | 156168 | 282290 | 155155 | 118120 | 229229 | 145 |
| 588 | Maitencillo | MA15 | 2 | L | 136172 | 156168 | 282290 | 155155 | 118120 | 229229 | 145 |
| 589 | Maitencillo | MA15 | 3 | H | 136172 | 168174 | 280294 | 155155 | 120122 | 229229 | 142 |
| 590 | Maitencillo | MA15 | 3 | B | 136172 | 168174 | 280294 | 155155 | 120122 | 229229 | 142 |
| 591 | Maitencillo | MA15 | 3 | M | 136172 | 168174 | 280294 | 155155 | 120122 | 229229 | 142 |
| 592 | Maitencillo | MA15 | 3 | L | 136172 | 168174 | 280294 | 155155 | 120122 | 229229 | 142 |
| 593 | Maitencillo | MA15 | 4 | H | 136172 | 168174 | 280294 | 155155 | 120122 | 229231 | 146 |
| 594 | Maitencillo | MA15 | 4 | B | 136172 | 168174 | 280294 | 155155 | 120122 | 229231 | 146 |
| 595 | Maitencillo | MA15 | 4 | M | 136172 | 168174 | 280294 | 155155 | 120122 | 229231 | 146 |
| 596 | Maitencillo | MA15 | 4 | L | 136172 | 168174 | 280294 | 155155 | 120122 | 229231 | 146 |
| 597 | Maitencillo | MA15 | 5 | H | 136172 | 168174 | 282294 | 155155 | 120122 | 229229 | 147 |
| 598 | Maitencillo | MA15 | 5 | B | 136172 | 160168 | 282290 | 155155 | 120122 | 229229 | 144 |
| 599 | Maitencillo | MA15 | 5 | M | 136172 | 160168 | 282290 | 155155 | 120122 | 229229 | 144 |
| 600 | Maitencillo | MA15 | 5 | L | 136172 | 160168 | 282290 | 155155 | 120122 | 229229 | 144 |

S1 Table. Continuation

| ♯ | Pop. | Nº of Plant | Replicate | Thallus zone | LESS1T3 | LESS1D4 | LESS1T9 | LESS1T11 | LESS2D22 | LESS2D25 | N° of genotype |
| --- | --- | --- | --- | --- | --- | --- | --- | --- | --- | --- | --- |
| 601 | LaPuntilla | LP1 | 1 | H | 148168 | 162166 | 278284 | 155189 | 117117 | 229229 | 148 |
| 602 | LaPuntilla | LP1 | 1 | B | 148168 | 162162 | 278284 | 155189 | 117117 | 229229 | 149 |
| 603 | LaPuntilla | LP1 | 1 | M | 148168 | 162162 | 278284 | 155189 | 117117 | 229229 | 149 |
| 604 | LaPuntilla | LP1 | 1 | L | 148168 | 162162 | 278284 | 155189 | 117117 | 229229 | 149 |
| 605 | LaPuntilla | LP1 | 2 | H | 148168 | 162162 | 278284 | 155189 | 117117 | 229229 | 149 |
| 606 | LaPuntilla | LP1 | 2 | B | 148168 | 162162 | 278284 | 155189 | 117117 | 229229 | 149 |
| 607 | LaPuntilla | LP1 | 2 | M | 148168 | 162162 | 278284 | 155189 | 117117 | 229229 | 149 |
| 608 | LaPuntilla | LP1 | 2 | L | 148168 | 162162 | 278284 | 155189 | 117117 | 229229 | 149 |
| 609 | LaPuntilla | LP1 | 3 | H | 148168 | 162166 | 278284 | 155189 | 117117 | 229229 | 148 |
| 610 | LaPuntilla | LP1 | 3 | B | 148168 | 162162 | 278284 | 155189 | 117117 | 229229 | 149 |
| 611 | LaPuntilla | LP1 | 3 | M | 148168 | 162162 | 278284 | 155189 | 117117 | 229229 | 149 |
| 612 | LaPuntilla | LP1 | 3 | L | 148168 | 162162 | 278284 | 155189 | 117117 | 229229 | 149 |
| 613 | LaPuntilla | LP1 | 4 | H | 148168 | 162162 | 278284 | 155189 | 117117 | 229229 | 149 |
| 614 | LaPuntilla | LP1 | 4 | B | 148168 | 162162 | 278284 | 155189 | 117117 | 229229 | 149 |
| 615 | LaPuntilla | LP1 | 4 | M | 148168 | 162162 | 278284 | 155189 | 117117 | 229229 | 149 |
| 616 | LaPuntilla | LP1 | 4 | L | 148168 | 162162 | 278284 | 155189 | 117117 | 229229 | 149 |
| 617 | LaPuntilla | LP1 | 5 | H | 148168 | 162166 | 278284 | 155189 | 117117 | 229229 | 148 |
| 618 | LaPuntilla | LP1 | 5 | B | 148168 | 162162 | 278284 | 155189 | 117117 | 229229 | 149 |
| 619 | LaPuntilla | LP1 | 5 | M | 148168 | 162162 | 278284 | 155189 | 117117 | 229229 | 149 |
| 620 | LaPuntilla | LP1 | 5 | L | 148168 | 162162 | 278284 | 155189 | 117117 | 229229 | 149 |
| 621 | LaPuntilla | LP2 | 1 | H | 148148 | 162162 | 270270 | 189189 | 117117 | 229229 | 150 |
| 622 | LaPuntilla | LP2 | 1 | B | 148148 | 162162 | 270270 | 189189 | 117117 | 229229 | 150 |
| 623 | LaPuntilla | LP2 | 1 | M | 148148 | 162162 | 270270 | 189189 | 117117 | 229229 | 150 |
| 624 | LaPuntilla | LP2 | 1 | L | 148148 | 162162 | 270270 | 189189 | 117117 | 229229 | 150 |
| 625 | LaPuntilla | LP2 | 2 | H | 148148 | 162166 | 270270 | 155189 | 117117 | 229229 | 151 |
| 626 | LaPuntilla | LP2 | 2 | B | 148148 | 162162 | 270270 | 155189 | 117117 | 229229 | 152 |
| 627 | LaPuntilla | LP2 | 2 | M | 148148 | 162162 | 270270 | 189189 | 117117 | 229229 | 150 |
| 628 | LaPuntilla | LP2 | 2 | L | 148148 | 162162 | 270270 | 189189 | 117117 | 229229 | 150 |
| 629 | LaPuntilla | LP2 | 3 | H | 148148 | 162162 | 270270 | 155189 | 117117 | 229229 | 152 |
| 630 | LaPuntilla | LP2 | 3 | B | 148148 | 162162 | 270270 | 155189 | 117117 | 229229 | 152 |
| 631 | LaPuntilla | LP2 | 3 | M | 148148 | 162162 | 270270 | 155189 | 117117 | 229229 | 152 |
| 632 | LaPuntilla | LP2 | 3 | L | 148148 | 162162 | 270270 | 155189 | 117117 | 229229 | 152 |
| 633 | LaPuntilla | LP2 | 4 | H | 148148 | 162166 | 270270 | 155189 | 117117 | 229229 | 151 |
| 634 | LaPuntilla | LP2 | 4 | B | 148148 | 162166 | 270270 | 155189 | 117117 | 229229 | 151 |
| 635 | LaPuntilla | LP2 | 4 | M | 148148 | 162162 | 270270 | 155189 | 117117 | 229229 | 152 |
| 636 | LaPuntilla | LP2 | 4 | L | 148148 | 162162 | 270270 | 155189 | 117117 | 229229 | 152 |
| 637 | LaPuntilla | LP2 | 5 | H | 148148 | 162162 | 270270 | 189189 | 117117 | 229229 | 150 |
| 638 | LaPuntilla | LP2 | 5 | B | 148148 | 162162 | 270270 | 189189 | 117117 | 229229 | 150 |
| 639 | LaPuntilla | LP2 | 5 | M | 148148 | 162162 | 270270 | 189189 | 117117 | 229229 | 150 |
| 640 | LaPuntilla | LP2 | 5 | L | 148148 | 162162 | 270270 | 189189 | 117117 | 229229 | 150 |
| 641 | LaPuntilla | LP3 | 1 | H | 148172 | 162166 | 280280 | 155189 | 117117 | 229245 | 153 |
| 642 | LaPuntilla | LP3 | 1 | B | 148172 | 162162 | 280280 | 155189 | 117117 | 229245 | 154 |
| 643 | LaPuntilla | LP3 | 1 | M | 148172 | 162162 | 280280 | 155189 | 117117 | 229245 | 154 |
| 644 | LaPuntilla | LP3 | 1 | L | 148172 | 162162 | 280280 | 155189 | 117117 | 229245 | 154 |
| 645 | LaPuntilla | LP3 | 2 | H | 148172 | 162166 | 280280 | 155189 | 117117 | 229245 | 153 |
| 646 | LaPuntilla | LP3 | 2 | B | 148172 | 162166 | 280280 | 155189 | 117117 | 229245 | 153 |
| 647 | LaPuntilla | LP3 | 2 | M | 148172 | 162162 | 280280 | 155189 | 117117 | 229245 | 154 |
| 648 | LaPuntilla | LP3 | 2 | L | 148172 | 162162 | 280280 | 155189 | 117117 | 229245 | 154 |
| 649 | LaPuntilla | LP3 | 3 | H | 148172 | 162166 | 280280 | 155189 | 117117 | 229245 | 153 |
| 650 | LaPuntilla | LP3 | 3 | B | 148172 | 162166 | 280280 | 155189 | 117117 | 229245 | 153 |
| 651 | LaPuntilla | LP3 | 3 | M | 148172 | 162162 | 280280 | 155189 | 117117 | 229245 | 154 |
| 652 | LaPuntilla | LP3 | 3 | L | 148172 | 162162 | 280280 | 155189 | 117117 | 229245 | 154 |
| 653 | LaPuntilla | LP3 | 4 | H | 148172 | 162162 | 280280 | 155189 | 117117 | 229245 | 154 |
| 654 | LaPuntilla | LP3 | 4 | B | 148172 | 162162 | 280280 | 155189 | 117117 | 229245 | 154 |
| 655 | LaPuntilla | LP3 | 4 | M | 148172 | 162162 | 280280 | 155189 | 117117 | 229245 | 154 |
| 656 | LaPuntilla | LP3 | 4 | L | 148172 | 162162 | 280280 | 155189 | 117117 | 229245 | 154 |
| 657 | LaPuntilla | LP3 | 5 | H | 148172 | 162166 | 280280 | 155189 | 117117 | 229245 | 152 |
| 658 | LaPuntilla | LP3 | 5 | B | 148172 | 162166 | 280280 | 155189 | 117117 | 229245 | 153 |
| 659 | LaPuntilla | LP3 | 5 | M | 148172 | 162162 | 280280 | 155189 | 117117 | 229245 | 154 |
| 660 | LaPuntilla | LP3 | 5 | L | 148172 | 162162 | 280280 | 155189 | 117117 | 229245 | 154 |
| 661 | LaPuntilla | LP4 | 1 | H | 148168 | 162164 | 278278 | 155189 | 117117 | 229229 | 155 |
| 662 | LaPuntilla | LP4 | 1 | B | 148168 | 162164 | 278278 | 155189 | 117117 | 229229 | 155 |
| 663 | LaPuntilla | LP4 | 1 | M | 148168 | 162164 | 278278 | 155189 | 117117 | 229229 | 155 |
| 664 | LaPuntilla | LP4 | 1 | L | 148168 | 162164 | 278278 | 155189 | 117117 | 229229 | 155 |
| 665 | LaPuntilla | LP4 | 2 | H | 148168 | 162164 | 278278 | 155189 | 117117 | 229229 | 155 |
| 666 | LaPuntilla | LP4 | 2 | B | 148168 | 162164 | 278278 | 155189 | 117117 | 229229 | 155 |
| 667 | LaPuntilla | LP4 | 2 | M | 148168 | 162164 | 278278 | 155189 | 117117 | 229229 | 155 |
| 668 | LaPuntilla | LP4 | 2 | L | 148168 | 162164 | 278278 | 155189 | 117117 | 229229 | 155 |
| 669 | LaPuntilla | LP4 | 3 | H | 148168 | 162164 | 278278 | 155189 | 117117 | 229229 | 155 |
| 670 | LaPuntilla | LP4 | 3 | B | 148168 | 162164 | 278278 | 155189 | 117117 | 229229 | 155 |
| 671 | LaPuntilla | LP4 | 3 | M | 148168 | 162164 | 278278 | 155189 | 117117 | 229229 | 155 |
| 672 | LaPuntilla | LP4 | 3 | L | 148168 | 162164 | 278278 | 155189 | 117117 | 229229 | 155 |
| 673 | LaPuntilla | LP4 | 4 | H | 148168 | 162164 | 278278 | 155189 | 117117 | 229229 | 155 |
| 674 | LaPuntilla | LP4 | 4 | B | 148168 | 162164 | 278278 | 155189 | 117117 | 229229 | 155 |
| 675 | LaPuntilla | LP4 | 4 | M | 148168 | 162164 | 278278 | 155189 | 117117 | 229229 | 155 |
| 676 | LaPuntilla | LP4 | 4 | L | 148168 | 162164 | 278278 | 155189 | 117117 | 229229 | 155 |
| 677 | LaPuntilla | LP4 | 5 | H | 148168 | 162164 | 278278 | 155189 | 117117 | 229229 | 155 |
| 678 | LaPuntilla | LP4 | 5 | B | 148168 | 162164 | 278278 | 155189 | 117117 | 229229 | 155 |
| 679 | LaPuntilla | LP4 | 5 | M | 148168 | 162164 | 278278 | 155189 | 117117 | 229229 | 155 |
| 680 | LaPuntilla | LP4 | 5 | L | 148168 | 162164 | 278278 | 155189 | 117117 | 229229 | 155 |
| 681 | LaPuntilla | LP5 | 1 | H | 158168 | 162164 | 280280 | 155189 | 117117 | 245245 | 156 |
| 682 | LaPuntilla | LP5 | 1 | B | 158168 | 162164 | 280280 | 155189 | 117117 | 245245 | 156 |
| 683 | LaPuntilla | LP5 | 1 | M | 158168 | 162164 | 280280 | 155189 | 117117 | 245245 | 156 |
| 684 | LaPuntilla | LP5 | 1 | L | 158168 | 162164 | 280280 | 155189 | 117117 | 245245 | 156 |
| 685 | LaPuntilla | LP5 | 2 | H | 148168 | 162162 | 278278 | 155155 | 117117 | 245245 | 157 |
| 686 | LaPuntilla | LP5 | 2 | B | 148168 | 162162 | 278278 | 155155 | 117117 | 245245 | 157 |
| 687 | LaPuntilla | LP5 | 2 | M | 148168 | 162162 | 278278 | 155155 | 117117 | 245245 | 157 |
| 688 | LaPuntilla | LP5 | 2 | L | 148168 | 162162 | 278278 | 155155 | 117117 | 245245 | 157 |
| 689 | LaPuntilla | LP5 | 3 | H | 148168 | 162166 | 278278 | 155155 | 117117 | 245245 | 158 |
| 690 | LaPuntilla | LP5 | 3 | B | 148168 | 162162 | 278278 | 155155 | 117117 | 245245 | 157 |
| 691 | LaPuntilla | LP5 | 3 | M | 148168 | 162162 | 278278 | 155155 | 117117 | 245245 | 157 |
| 692 | LaPuntilla | LP5 | 3 | L | 148168 | 162162 | 278278 | 155155 | 117117 | 245245 | 157 |
| 693 | LaPuntilla | LP5 | 4 | H | 148168 | 162162 | 278278 | 155155 | 117117 | 245245 | 157 |
| 694 | LaPuntilla | LP5 | 4 | B | 148168 | 162162 | 278278 | 155155 | 117117 | 245245 | 157 |
| 695 | LaPuntilla | LP5 | 4 | M | 148168 | 162162 | 278278 | 155155 | 117117 | 245245 | 157 |
| 696 | LaPuntilla | LP5 | 4 | L | 148168 | 162162 | 278278 | 155155 | 117117 | 245245 | 157 |
| 697 | LaPuntilla | LP5 | 5 | H | 148168 | 162162 | 278278 | 155155 | 117117 | 245245 | 157 |
| 698 | LaPuntilla | LP5 | 5 | B | 148168 | 162162 | 278278 | 155155 | 117117 | 245245 | 157 |
| 699 | LaPuntilla | LP5 | 5 | M | 148168 | 162162 | 278278 | 155155 | 117117 | 245245 | 157 |
| 700 | LaPuntilla | LP5 | 5 | L | 148168 | 162162 | 278278 | 155155 | 117117 | 245245 | 157 |

S1 Table. Continuation

| ♯ | Pop. | Nº of Plant | Replicate | Thallus zone | LESS1T3 | LESS1D4 | LESS1T9 | LESS1T11 | LESS2D22 | LESS2D25 | N° of genotype |
| --- | --- | --- | --- | --- | --- | --- | --- | --- | --- | --- | --- |
| 701 | LaPuntilla | LP6 | 1 | H | 148172 | 162162 | 280280 | 155189 | 117117 | 229247 | 159 |
| 702 | LaPuntilla | LP6 | 1 | B | 148172 | 162162 | 280280 | 189189 | 117117 | 229247 | 160 |
| 703 | LaPuntilla | LP6 | 1 | M | 148172 | 162162 | 280280 | 189189 | 117117 | 229247 | 160 |
| 704 | LaPuntilla | LP6 | 1 | L | 148172 | 162162 | 280280 | 189189 | 117117 | 229247 | 160 |
| 705 | LaPuntilla | LP6 | 2 | H | 148172 | 162162 | 280280 | 189189 | 117117 | 229247 | 160 |
| 706 | LaPuntilla | LP6 | 2 | B | 148172 | 162162 | 280280 | 189189 | 117117 | 229247 | 160 |
| 707 | LaPuntilla | LP6 | 2 | M | 148172 | 162162 | 280280 | 189189 | 117117 | 229247 | 160 |
| 708 | LaPuntilla | LP6 | 2 | L | 148172 | 162162 | 280280 | 189189 | 117117 | 229247 | 160 |
| 709 | LaPuntilla | LP6 | 3 | H | 148172 | 162162 | 280280 | 189189 | 117117 | 229247 | 160 |
| 710 | LaPuntilla | LP6 | 3 | B | 148172 | 162162 | 280280 | 189189 | 117117 | 229247 | 160 |
| 711 | LaPuntilla | LP6 | 3 | M | 148172 | 162162 | 280280 | 189189 | 117117 | 229247 | 160 |
| 712 | LaPuntilla | LP6 | 3 | L | 148172 | 162162 | 280280 | 189189 | 117117 | 229247 | 160 |
| 713 | LaPuntilla | LP6 | 4 | H | 148172 | 162162 | 280280 | 155189 | 117117 | 229247 | 159 |
| 714 | LaPuntilla | LP6 | 4 | B | 148172 | 162162 | 280280 | 189189 | 117117 | 229247 | 160 |
| 715 | LaPuntilla | LP6 | 4 | M | 148172 | 162162 | 280280 | 189189 | 117117 | 229247 | 160 |
| 716 | LaPuntilla | LP6 | 4 | L | 148172 | 162162 | 280280 | 189189 | 117117 | 229247 | 160 |
| 717 | LaPuntilla | LP6 | 5 | H | 148168 | 162162 | 278284 | 155189 | 117117 | 229229 | 149 |
| 718 | LaPuntilla | LP6 | 5 | B | 148168 | 162162 | 278284 | 155189 | 117117 | 229229 | 149 |
| 719 | LaPuntilla | LP6 | 5 | M | 148168 | 162162 | 278284 | 155189 | 117117 | 229229 | 149 |
| 720 | LaPuntilla | LP6 | 5 | L | 148168 | 162162 | 278284 | 155189 | 117117 | 229229 | 149 |
| 721 | LaPuntilla | LP7 | 1 | H | 148148 | 162162 | 278278 | 155155 | 117117 | 229229 | 161 |
| 722 | LaPuntilla | LP7 | 1 | B | 148148 | 162162 | 278278 | 155155 | 117117 | 229229 | 161 |
| 723 | LaPuntilla | LP7 | 1 | M | 148148 | 162162 | 278278 | 155155 | 117117 | 229229 | 161 |
| 724 | LaPuntilla | LP7 | 1 | L | 148148 | 162162 | 278278 | 155155 | 117117 | 229229 | 161 |
| 725 | LaPuntilla | LP7 | 2 | H | 148168 | 164166 | 280280 | 155155 | 117117 | 229247 | 162 |
| 726 | LaPuntilla | LP7 | 2 | B | 148168 | 162162 | 280280 | 155155 | 117117 | 229247 | 163 |
| 727 | LaPuntilla | LP7 | 2 | M | 148168 | 162162 | 280280 | 155155 | 117117 | 229247 | 163 |
| 728 | LaPuntilla | LP7 | 2 | L | 148168 | 162162 | 280280 | 155155 | 117117 | 229247 | 163 |
| 729 | LaPuntilla | LP7 | 3 | H | 148168 | 162162 | 280280 | 155155 | 117117 | 229247 | 163 |
| 730 | LaPuntilla | LP7 | 3 | B | 148168 | 162162 | 280280 | 155155 | 117117 | 229247 | 163 |
| 731 | LaPuntilla | LP7 | 3 | M | 148168 | 162162 | 280280 | 155155 | 117117 | 229247 | 163 |
| 732 | LaPuntilla | LP7 | 3 | L | 148168 | 162162 | 280280 | 155155 | 117117 | 229247 | 163 |
| 733 | LaPuntilla | LP7 | 4 | H | 148168 | 162162 | 280280 | 155155 | 117117 | 229247 | 163 |
| 734 | LaPuntilla | LP7 | 4 | B | 148168 | 162162 | 280280 | 155155 | 117117 | 229247 | 163 |
| 735 | LaPuntilla | LP7 | 4 | M | 148168 | 162162 | 280280 | 155155 | 117117 | 229247 | 163 |
| 736 | LaPuntilla | LP7 | 4 | L | 148168 | 162162 | 280280 | 155155 | 117117 | 229247 | 163 |
| 737 | LaPuntilla | LP7 | 5 | H | 148168 | 162162 | 280280 | 155155 | 117117 | 229247 | 163 |
| 738 | LaPuntilla | LP7 | 5 | B | 148168 | 162162 | 280280 | 155155 | 117117 | 229247 | 163 |
| 739 | LaPuntilla | LP7 | 5 | M | 148168 | 162162 | 280280 | 155155 | 117117 | 229247 | 163 |
| 740 | LaPuntilla | LP7 | 5 | L | 148168 | 162162 | 280280 | 155155 | 117117 | 229247 | 163 |
| 741 | LaPuntilla | LP8 | 1 | H | 168168 | 162162 | 280280 | 155155 | 117117 | 229245 | 164 |
| 742 | LaPuntilla | LP8 | 1 | B | 168168 | 162162 | 280280 | 155155 | 117117 | 229245 | 164 |
| 743 | LaPuntilla | LP8 | 1 | M | 168168 | 162162 | 280280 | 155155 | 117117 | 229245 | 164 |
| 744 | LaPuntilla | LP8 | 1 | L | 168168 | 162162 | 280280 | 155155 | 117117 | 229245 | 164 |
| 745 | LaPuntilla | LP8 | 2 | H | 168168 | 162162 | 280280 | 155155 | 117117 | 229245 | 164 |
| 746 | LaPuntilla | LP8 | 2 | B | 168168 | 162162 | 280280 | 155155 | 117117 | 229245 | 164 |
| 747 | LaPuntilla | LP8 | 2 | M | 168168 | 162162 | 280280 | 155155 | 117117 | 229245 | 164 |
| 748 | LaPuntilla | LP8 | 2 | L | 168168 | 162162 | 280280 | 155155 | 117117 | 229245 | 164 |
| 749 | LaPuntilla | LP8 | 3 | H | 168168 | 162162 | 280280 | 155155 | 117117 | 229245 | 164 |
| 750 | LaPuntilla | LP8 | 3 | B | 168168 | 162162 | 280280 | 155155 | 117117 | 229245 | 164 |
| 751 | LaPuntilla | LP8 | 3 | M | 168168 | 162162 | 280280 | 155155 | 117117 | 229245 | 164 |
| 752 | LaPuntilla | LP8 | 3 | L | 168168 | 162162 | 280280 | 155155 | 117117 | 229245 | 164 |
| 753 | LaPuntilla | LP8 | 4 | H | 168168 | 162162 | 280280 | 155155 | 117117 | 229245 | 164 |
| 754 | LaPuntilla | LP8 | 4 | B | 168168 | 162162 | 280280 | 155155 | 117117 | 229245 | 164 |
| 755 | LaPuntilla | LP8 | 4 | M | 168168 | 162162 | 280280 | 155155 | 117117 | 229245 | 164 |
| 756 | LaPuntilla | LP8 | 4 | L | 168168 | 162162 | 280280 | 155155 | 117117 | 229245 | 164 |
| 757 | LaPuntilla | LP8 | 5 | H | 168168 | 162162 | 280280 | 155155 | 117117 | 229245 | 164 |
| 758 | LaPuntilla | LP8 | 5 | B | 168168 | 162162 | 280280 | 155155 | 117117 | 229245 | 164 |
| 759 | LaPuntilla | LP8 | 5 | M | 168168 | 162162 | 280280 | 155155 | 117117 | 229245 | 164 |
| 760 | LaPuntilla | LP8 | 5 | L | 168168 | 162162 | 280280 | 155155 | 117117 | 229245 | 164 |
| 761 | LaPuntilla | LP9 | 1 | H | 148168 | 162162 | 278284 | 155191 | 117117 | 245245 | 165 |
| 762 | LaPuntilla | LP9 | 1 | B | 148168 | 162162 | 278284 | 155191 | 117117 | 245245 | 165 |
| 763 | LaPuntilla | LP9 | 1 | M | 148168 | 162162 | 278284 | 155191 | 117117 | 245245 | 165 |
| 764 | LaPuntilla | LP9 | 1 | L | 148168 | 162162 | 278284 | 155191 | 117117 | 245245 | 165 |
| 765 | LaPuntilla | LP9 | 2 | H | 148168 | 162162 | 278284 | 155191 | 117117 | 245245 | 165 |
| 766 | LaPuntilla | LP9 | 2 | B | 148168 | 162162 | 278284 | 155191 | 117117 | 245245 | 165 |
| 767 | LaPuntilla | LP9 | 2 | M | 148168 | 162162 | 278284 | 155191 | 117117 | 245245 | 165 |
| 768 | LaPuntilla | LP9 | 2 | L | 148168 | 162162 | 278284 | 155189 | 117117 | 245245 | 166 |
| 769 | LaPuntilla | LP9 | 3 | H | 148168 | 162162 | 278284 | 155189 | 117117 | 245245 | 166 |
| 770 | LaPuntilla | LP9 | 3 | B | 148168 | 162162 | 278284 | 155191 | 117117 | 245245 | 165 |
| 771 | LaPuntilla | LP9 | 3 | M | 148168 | 162162 | 278284 | 155191 | 117117 | 245245 | 165 |
| 772 | LaPuntilla | LP9 | 3 | L | 148168 | 162162 | 278284 | 155191 | 117117 | 245245 | 165 |
| 773 | LaPuntilla | LP9 | 4 | H | 148168 | 162162 | 278284 | 155191 | 117117 | 245245 | 165 |
| 774 | LaPuntilla | LP9 | 4 | B | 148168 | 162162 | 278284 | 155191 | 117117 | 245245 | 165 |
| 775 | LaPuntilla | LP9 | 4 | M | 148168 | 162162 | 278284 | 155191 | 117117 | 245245 | 165 |
| 776 | LaPuntilla | LP9 | 4 | L | 148168 | 162162 | 278284 | 155191 | 117117 | 245245 | 165 |
| 777 | LaPuntilla | LP9 | 5 | H | 148168 | 162162 | 278284 | 155191 | 117117 | 245245 | 165 |
| 778 | LaPuntilla | LP9 | 5 | B | 148168 | 162162 | 278284 | 155191 | 117117 | 245245 | 165 |
| 779 | LaPuntilla | LP9 | 5 | M | 148168 | 162162 | 278284 | 155191 | 117117 | 245245 | 165 |
| 780 | LaPuntilla | LP9 | 5 | L | 148168 | 162162 | 278284 | 155191 | 117117 | 245245 | 165 |
| 781 | LaPuntilla | LP10 | 1 | H | 148148 | 162162 | 280280 | 177189 | 117117 | 229247 | 167 |
| 782 | LaPuntilla | LP10 | 1 | B | 148148 | 162162 | 280280 | 177189 | 117117 | 229247 | 167 |
| 783 | LaPuntilla | LP10 | 1 | M | 148148 | 162162 | 280280 | 177189 | 117117 | 229247 | 167 |
| 784 | LaPuntilla | LP10 | 1 | L | 148148 | 162162 | 280280 | 177189 | 117117 | 229247 | 167 |
| 785 | LaPuntilla | LP10 | 2 | H | 148148 | 162162 | 280280 | 177189 | 117117 | 229247 | 167 |
| 786 | LaPuntilla | LP10 | 2 | B | 148148 | 162162 | 280280 | 177189 | 117117 | 229247 | 167 |
| 787 | LaPuntilla | LP10 | 2 | M | 148148 | 162162 | 280280 | 177189 | 117117 | 229247 | 167 |
| 788 | LaPuntilla | LP10 | 2 | L | 148148 | 162162 | 280280 | 177189 | 117117 | 229247 | 167 |
| 789 | LaPuntilla | LP10 | 3 | H | 148148 | 162162 | 280280 | 177189 | 117117 | 229247 | 167 |
| 790 | LaPuntilla | LP10 | 3 | B | 148148 | 162162 | 280280 | 177189 | 117117 | 229247 | 167 |
| 791 | LaPuntilla | LP10 | 3 | M | 148148 | 162162 | 280280 | 177189 | 117117 | 229247 | 167 |
| 792 | LaPuntilla | LP10 | 3 | L | 148148 | 162162 | 280280 | 177189 | 117117 | 229247 | 167 |
| 793 | LaPuntilla | LP10 | 4 | H | 148148 | 162162 | 280280 | 177189 | 117117 | 229247 | 167 |
| 794 | LaPuntilla | LP10 | 4 | B | 148148 | 162162 | 280280 | 177189 | 117117 | 229247 | 167 |
| 795 | LaPuntilla | LP10 | 4 | M | 148148 | 162162 | 280280 | 177189 | 117117 | 229247 | 167 |
| 796 | LaPuntilla | LP10 | 4 | L | 148148 | 162162 | 280280 | 177189 | 117117 | 229247 | 167 |
| 797 | LaPuntilla | LP10 | 5 | H | 148148 | 162162 | 280280 | 177189 | 117117 | 229247 | 167 |
| 798 | LaPuntilla | LP10 | 5 | B | 148148 | 162162 | 280280 | 177189 | 117117 | 229247 | 167 |
| 799 | LaPuntilla | LP10 | 5 | M | 148148 | 162162 | 280280 | 177189 | 117117 | 229247 | 167 |
| 800 | LaPuntilla | LP10 | 5 | L | 148148 | 162162 | 280280 | 177189 | 117117 | 229247 | 167 |

S1 Table. Continuation

| ♯ | Pop. | Nº of Plant | Replicate | Thallus zone | LESS1T3 | LESS1D4 | LESS1T9 | LESS1T11 | LESS2D22 | LESS2D25 | N° of genotype |
| --- | --- | --- | --- | --- | --- | --- | --- | --- | --- | --- | --- |
| 801 | LaPuntilla | LP11 | 1 | H | 148168 | 162162 | 270270 | 155155 | 117117 | 229229 | 168 |
| 802 | LaPuntilla | LP11 | 1 | B | 148168 | 162162 | 270270 | 155155 | 117117 | 229229 | 168 |
| 803 | LaPuntilla | LP11 | 1 | M | 148168 | 162162 | 270270 | 155155 | 117117 | 229229 | 168 |
| 804 | LaPuntilla | LP11 | 1 | L | 148168 | 162162 | 270270 | 155155 | 117117 | 229229 | 168 |
| 805 | LaPuntilla | LP11 | 2 | H | 148168 | 162162 | 280280 | 155155 | 117117 | 229247 | 163 |
| 806 | LaPuntilla | LP11 | 2 | B | 148168 | 162162 | 270280 | 155155 | 117117 | 229229 | 169 |
| 807 | LaPuntilla | LP11 | 2 | M | 148168 | 162162 | 270270 | 155155 | 117117 | 229229 | 168 |
| 808 | LaPuntilla | LP11 | 2 | L | 148168 | 162162 | 270270 | 155155 | 117117 | 229229 | 168 |
| 809 | LaPuntilla | LP11 | 3 | H | 148168 | 162162 | 270270 | 155155 | 117117 | 229229 | 168 |
| 810 | LaPuntilla | LP11 | 3 | B | 148168 | 162162 | 270270 | 155155 | 117117 | 229229 | 168 |
| 811 | LaPuntilla | LP11 | 3 | M | 148168 | 162162 | 270270 | 155155 | 117117 | 229229 | 168 |
| 812 | LaPuntilla | LP11 | 3 | L | 148168 | 162162 | 270270 | 155155 | 117117 | 229229 | 168 |
| 813 | LaPuntilla | LP11 | 4 | H | 148168 | 162162 | 270270 | 155155 | 117117 | 229229 | 168 |
| 814 | LaPuntilla | LP11 | 4 | B | 148168 | 162162 | 270270 | 155155 | 117117 | 229229 | 168 |
| 815 | LaPuntilla | LP11 | 4 | M | 148168 | 162162 | 270270 | 155155 | 117117 | 229229 | 168 |
| 816 | LaPuntilla | LP11 | 4 | L | 148168 | 162162 | 270270 | 155155 | 117117 | 229229 | 168 |
| 817 | LaPuntilla | LP11 | 5 | H | 148168 | 162162 | 270270 | 155155 | 117117 | 229229 | 168 |
| 818 | LaPuntilla | LP11 | 5 | B | 148168 | 162162 | 270270 | 155155 | 117117 | 229229 | 168 |
| 819 | LaPuntilla | LP11 | 5 | M | 148168 | 162162 | 270270 | 155155 | 117117 | 229229 | 168 |
| 820 | LaPuntilla | LP11 | 5 | L | 148168 | 162162 | 270270 | 155155 | 117117 | 229229 | 168 |
| 821 | LaPuntilla | LP12 | 1 | H | 148148 | 162162 | 280280 | 155189 | 117117 | 229245 | 170 |
| 822 | LaPuntilla | LP12 | 1 | B | 148148 | 162162 | 280280 | 155189 | 117117 | 229245 | 170 |
| 823 | LaPuntilla | LP12 | 1 | M | 148148 | 162162 | 280280 | 155189 | 117117 | 229245 | 170 |
| 824 | LaPuntilla | LP12 | 1 | L | 148148 | 162162 | 280280 | 155189 | 117117 | 229245 | 170 |
| 825 | LaPuntilla | LP12 | 2 | H | 148148 | 162162 | 280280 | 155189 | 117117 | 229245 | 170 |
| 826 | LaPuntilla | LP12 | 2 | B | 148148 | 162162 | 280280 | 155189 | 117117 | 229245 | 170 |
| 827 | LaPuntilla | LP12 | 2 | M | 148148 | 162162 | 280280 | 155189 | 117117 | 229245 | 170 |
| 828 | LaPuntilla | LP12 | 2 | L | 148148 | 162162 | 280280 | 155189 | 117117 | 229245 | 170 |
| 829 | LaPuntilla | LP12 | 3 | H | 148148 | 162162 | 280280 | 155189 | 117117 | 229245 | 170 |
| 830 | LaPuntilla | LP12 | 3 | B | 148148 | 162162 | 280280 | 155189 | 117117 | 229245 | 170 |
| 831 | LaPuntilla | LP12 | 3 | M | 148148 | 162162 | 280280 | 155189 | 117117 | 229245 | 170 |
| 832 | LaPuntilla | LP12 | 3 | L | 148148 | 162162 | 280280 | 155189 | 117117 | 229245 | 170 |
| 833 | LaPuntilla | LP12 | 4 | H | 148148 | 162162 | 280280 | 155189 | 117117 | 229245 | 170 |
| 834 | LaPuntilla | LP12 | 4 | B | 148148 | 162162 | 280280 | 155189 | 117117 | 229245 | 170 |
| 835 | LaPuntilla | LP12 | 4 | M | 148148 | 162162 | 280280 | 155189 | 117117 | 229245 | 170 |
| 836 | LaPuntilla | LP12 | 4 | L | 148148 | 162162 | 280280 | 155189 | 117117 | 229245 | 170 |
| 837 | LaPuntilla | LP12 | 5 | H | 148148 | 162162 | 280280 | 155189 | 117117 | 229245 | 170 |
| 838 | LaPuntilla | LP12 | 5 | B | 148148 | 162162 | 280280 | 155189 | 117117 | 229245 | 170 |
| 839 | LaPuntilla | LP12 | 5 | M | 148148 | 162162 | 280280 | 155189 | 117117 | 229245 | 170 |
| 840 | LaPuntilla | LP12 | 5 | L | 148148 | 162162 | 280280 | 155189 | 117117 | 229245 | 170 |
| 841 | LaPuntilla | LP13 | 1 | H | 148172 | 162166 | 280280 | 155189 | 117117 | 229245 | 153 |
| 842 | LaPuntilla | LP13 | 1 | B | 148172 | 162162 | 280280 | 155189 | 117117 | 229245 | 154 |
| 843 | LaPuntilla | LP13 | 1 | M | 148172 | 162162 | 280280 | 155189 | 117117 | 229245 | 154 |
| 844 | LaPuntilla | LP13 | 1 | L | 148172 | 162162 | 280280 | 155189 | 117117 | 229245 | 154 |
| 845 | LaPuntilla | LP13 | 2 | H | 148172 | 162162 | 280280 | 155189 | 117117 | 229245 | 154 |
| 846 | LaPuntilla | LP13 | 2 | B | 148172 | 162162 | 280280 | 155189 | 117117 | 229245 | 154 |
| 847 | LaPuntilla | LP13 | 2 | M | 148172 | 162162 | 280280 | 155189 | 117117 | 229245 | 154 |
| 848 | LaPuntilla | LP13 | 2 | L | 148172 | 162162 | 280280 | 155189 | 117117 | 229245 | 154 |
| 849 | LaPuntilla | LP13 | 3 | H | 148172 | 162162 | 280280 | 155189 | 117117 | 229245 | 154 |
| 850 | LaPuntilla | LP13 | 3 | B | 148172 | 162162 | 280280 | 155189 | 117117 | 229245 | 154 |
| 851 | LaPuntilla | LP13 | 3 | M | 148172 | 162162 | 280280 | 155189 | 117117 | 229245 | 154 |
| 852 | LaPuntilla | LP13 | 3 | L | 148172 | 162162 | 280280 | 155189 | 117117 | 229245 | 154 |
| 853 | LaPuntilla | LP13 | 4 | H | 148172 | 162162 | 280280 | 155189 | 117117 | 229245 | 154 |
| 854 | LaPuntilla | LP13 | 4 | B | 148172 | 162162 | 280280 | 155189 | 117117 | 229245 | 154 |
| 855 | LaPuntilla | LP13 | 4 | M | 148172 | 162162 | 280280 | 155189 | 117117 | 229245 | 154 |
| 856 | LaPuntilla | LP13 | 4 | L | 148172 | 162162 | 280280 | 155189 | 117117 | 229245 | 154 |
| 857 | LaPuntilla | LP13 | 5 | H | 148172 | 162162 | 280280 | 155189 | 117117 | 229245 | 154 |
| 858 | LaPuntilla | LP13 | 5 | B | 148172 | 162162 | 280280 | 155189 | 117117 | 229245 | 154 |
| 859 | LaPuntilla | LP13 | 5 | M | 148172 | 162162 | 280280 | 155189 | 117117 | 229245 | 154 |
| 860 | LaPuntilla | LP13 | 5 | L | 148172 | 162162 | 280280 | 155189 | 117117 | 229245 | 154 |
| 861 | LaPuntilla | LP14 | 1 | H | 148148 | 162162 | 278284 | 155155 | 117117 | 229245 | 171 |
| 862 | LaPuntilla | LP14 | 1 | B | 148148 | 162162 | 278284 | 155155 | 117117 | 229245 | 171 |
| 863 | LaPuntilla | LP14 | 1 | M | 148148 | 162162 | 278284 | 155155 | 117117 | 229245 | 171 |
| 864 | LaPuntilla | LP14 | 1 | L | 148148 | 162162 | 278284 | 155155 | 117117 | 229245 | 171 |
| 865 | LaPuntilla | LP14 | 2 | H | 148148 | 162162 | 278284 | 155155 | 117117 | 229245 | 171 |
| 866 | LaPuntilla | LP14 | 2 | B | 148148 | 162162 | 278284 | 155155 | 117117 | 229245 | 171 |
| 867 | LaPuntilla | LP14 | 2 | M | 148148 | 162162 | 278284 | 155155 | 117117 | 229245 | 171 |
| 868 | LaPuntilla | LP14 | 2 | L | 148148 | 162162 | 278284 | 155155 | 117117 | 229245 | 171 |
| 869 | LaPuntilla | LP14 | 3 | H | 148148 | 162162 | 278284 | 155155 | 117117 | 229245 | 171 |
| 870 | LaPuntilla | LP14 | 3 | B | 148148 | 162162 | 278284 | 155155 | 117117 | 229245 | 171 |
| 871 | LaPuntilla | LP14 | 3 | M | 148148 | 162162 | 278284 | 155155 | 117117 | 229245 | 171 |
| 872 | LaPuntilla | LP14 | 3 | L | 148148 | 162162 | 278284 | 155155 | 117117 | 229245 | 171 |
| 873 | LaPuntilla | LP14 | 4 | H | 148148 | 162162 | 278284 | 155155 | 117117 | 229245 | 171 |
| 874 | LaPuntilla | LP14 | 4 | B | 148148 | 162162 | 278284 | 155155 | 117117 | 229245 | 171 |
| 875 | LaPuntilla | LP14 | 4 | M | 148148 | 162162 | 278284 | 155155 | 117117 | 229245 | 171 |
| 876 | LaPuntilla | LP14 | 4 | L | 148148 | 162162 | 278284 | 155155 | 117117 | 229245 | 171 |
| 877 | LaPuntilla | LP14 | 5 | H | 148148 | 162162 | 278284 | 155155 | 117117 | 229245 | 171 |
| 878 | LaPuntilla | LP14 | 5 | B | 148148 | 162162 | 278284 | 155155 | 117117 | 229245 | 171 |
| 879 | LaPuntilla | LP14 | 5 | M | 148148 | 162162 | 278284 | 155155 | 117117 | 229245 | 171 |
| 880 | LaPuntilla | LP14 | 5 | L | 148148 | 162162 | 278284 | 155155 | 117117 | 229245 | 171 |
| 881 | LaPuntilla | LP15 | 1 | H | 148168 | 162162 | 270270 | 155155 | 117117 | 229229 | 168 |
| 882 | LaPuntilla | LP15 | 1 | B | 148168 | 162162 | 270270 | 155155 | 117117 | 229229 | 168 |
| 883 | LaPuntilla | LP15 | 1 | M | 148168 | 162162 | 270270 | 155155 | 117117 | 229229 | 168 |
| 884 | LaPuntilla | LP15 | 1 | L | 148168 | 162162 | 270270 | 155155 | 117117 | 229229 | 168 |
| 885 | LaPuntilla | LP15 | 2 | H | 148168 | 162162 | 270270 | 155155 | 117117 | 229229 | 168 |
| 886 | LaPuntilla | LP15 | 2 | B | 148168 | 162162 | 270270 | 155155 | 117117 | 229229 | 168 |
| 887 | LaPuntilla | LP15 | 2 | M | 148168 | 162162 | 270270 | 155155 | 117117 | 229229 | 168 |
| 888 | LaPuntilla | LP15 | 2 | L | 148168 | 162162 | 270270 | 155155 | 117117 | 229229 | 168 |
| 889 | LaPuntilla | LP15 | 3 | H | 148168 | 162162 | 270270 | 155155 | 117117 | 229229 | 168 |
| 890 | LaPuntilla | LP15 | 3 | B | 148168 | 162162 | 270270 | 155155 | 117117 | 229229 | 168 |
| 891 | LaPuntilla | LP15 | 3 | M | 148168 | 162162 | 270270 | 155155 | 117117 | 229229 | 168 |
| 892 | LaPuntilla | LP15 | 3 | L | 148168 | 162162 | 270270 | 155155 | 117117 | 229229 | 168 |
| 893 | LaPuntilla | LP15 | 4 | H | 148168 | 162162 | 270270 | 155155 | 117117 | 229229 | 168 |
| 894 | LaPuntilla | LP15 | 4 | B | 148168 | 162162 | 270270 | 155155 | 117117 | 229229 | 168 |
| 895 | LaPuntilla | LP15 | 4 | M | 148168 | 162162 | 270270 | 155155 | 117117 | 229229 | 168 |
| 896 | LaPuntilla | LP15 | 4 | L | 148168 | 162162 | 270270 | 155155 | 117117 | 229229 | 168 |
| 897 | LaPuntilla | LP15 | 5 | H | 148168 | 162162 | 270270 | 155155 | 117117 | 229229 | 168 |
| 898 | LaPuntilla | LP15 | 5 | B | 148168 | 162162 | 270270 | 155155 | 117117 | 229229 | 168 |
| 899 | LaPuntilla | LP15 | 5 | M | 148168 | 162162 | 270270 | 155155 | 117117 | 229229 | 168 |
| 900 | LaPuntilla | LP15 | 5 | L | 148168 | 162162 | 270270 | 155155 | 117117 | 229229 | 168 |
